# SARS-CoV-2 aberrantly elevates mitochondrial bioenergetics to induce robust virus propagation

**DOI:** 10.1038/s41392-024-01836-x

**Published:** 2024-05-11

**Authors:** Hye Jin Shin, Wooseong Lee, Keun Bon Ku, Gun Young Yoon, Hyun-Woo Moon, Chonsaeng Kim, Mi-Hwa Kim, Yoon-Sun Yi, Sangmi Jun, Bum-Tae Kim, Jong-Won Oh, Aleem Siddiqui, Seong-Jun Kim

**Affiliations:** 1 Department of Convergent Research of Emerging Virus Infection, Korea Research Institute of Chemical Technology, Daejeon, 34114 Republic of Korea; 2 Gyeongnam Biohealth Research Center, Gyeongnam Branch Institute, Korea Institute of Toxicology, Jinju, 52834 Republic of Korea; 3 Center for Research Equipment, Korea Basic Science Institute, Cheongju, Chungcheongbuk-do 28119 Republic of Korea; 4 Department of Biotechnology, Yonsei University, Seoul, 03722 Republic of Korea; 5 Division of Infectious Diseases, School of Medicine, University of California, San Diego, La Jolla, CA 92093 USA; 6 Present Address: Department of Microbiology, Chungnam National University School of Medicine, Daejeon, 35015 Republic of Korea

**Keywords:** Drug screening, Infection

## Abstract

Severe acute respiratory syndrome coronavirus 2 (SARS-CoV-2) is a ‘highly transmissible respiratory pathogen, leading to severe multi-organ damage. However, knowledge regarding SARS-CoV-2-induced cellular alterations is limited. In this study, we report that SARS-CoV-2 aberrantly elevates mitochondrial bioenergetics and activates the EGFR-mediated cell survival signal cascade during the early stage of viral infection. SARS-CoV-2 causes an increase in mitochondrial transmembrane potential via the SARS-CoV-2 RNA-nucleocapsid cluster, thereby abnormally promoting mitochondrial elongation and the OXPHOS process, followed by enhancing ATP production. Furthermore, SARS-CoV-2 activates the EGFR signal cascade and subsequently induces mitochondrial EGFR trafficking, contributing to abnormal OXPHOS process and viral propagation. Approved EGFR inhibitors remarkably reduce SARS-CoV-2 propagation, among which vandetanib exhibits the highest antiviral efficacy. Treatment of SARS-CoV-2-infected cells with vandetanib decreases SARS-CoV-2-induced EGFR trafficking to the mitochondria and restores SARS-CoV-2-induced aberrant elevation in OXPHOS process and ATP generation, thereby resulting in the reduction of SARS-CoV-2 propagation. Furthermore, oral administration of vandetanib to SARS-CoV-2-infected hACE2 transgenic mice reduces SARS-CoV-2 propagation in lung tissue and mitigates SARS-CoV-2-induced lung inflammation. Vandetanib also exhibits potent antiviral activity against various SARS-CoV-2 variants of concern, including alpha, beta, delta and omicron, in in vitro cell culture experiments. Taken together, our findings provide novel insight into SARS-CoV-2-induced alterations in mitochondrial dynamics and EGFR trafficking during the early stage of viral infection and their roles in robust SARS-CoV-2 propagation, suggesting that EGFR is an attractive host target for combating COVID-19.

## Introduction

Severe acute respiratory syndrome coronavirus 2 (SARS-CoV-2) poses a global threat, causing severe respiratory illness and fatalities.^1,2^ Phylogenetically related to the 2003 SARS-CoV epidemic,^3,4^ SARS-CoV-2 is an enveloped, single-stranded positive-sense RNA virus in the *Coronaviridae* family.^5^ Utilising host factors angiotensin-converting enzyme carboxypeptidase (ACE2) and transmembrane serine protease 2 (TMPRSS2), SARS-CoV-2 enters cells and employs its RNA-dependent RNA polymerase (RdRp) for genome replication.^6–8^ Upon ACE2-mediated endocytosis, the SARS-CoV-2 nucleocapsid protein forms a complex with viral RNA (SARS-CoV-2 RNA-nucleocapsid cluster) during cellular entry.^9,10^

Viruses manipulate various intracellular events, such as endoplasmic reticulum (ER) stress, oxidative stress and Ca^2+^ flux between the ER and the mitochondria, influencing viral propagation and inflammation.^11–14^ Notably, virus-induced mitochondrial Ca^2+^ overload can compromise mitochondrial membrane potential (ΔΨm), a critical step in regulating mitochondrial dynamics for cellular homoeostasis.^15–18^ Mitochondrial dynamics are crucial for the maintenance of cellular homoeostasis and are tightly regulated to overcome stress conditions.^19^ The morphology of mitochondria is altered by two opposing processes: fusion and fission.^20^ Mitochondrial fusion involves mitofusins (MFN1 and 2) and optic atrophy 1 (OPA1). Mitochondrial fission is mediated by the recruitment of dynamin-related protein 1 (Drp1), which is regulated by phosphorylation (S616) and dephosphorylation (S637).^21^ For several mechanisms underlying viral replication and the evasion of antiviral innate immune signalling, mitochondrial fusion is required.^22,23^ Recent evidence suggests that virus infections induce abnormal mitochondrial dynamics and perturb mitochondrial bioenergetics, crucial for viral propagation and the antiviral innate immune response.^24,25^

Homoeostasis regulation in host cells is vital for coronaviruses.^26^ The pro-survival role of epidermal growth factor receptors (EGFR), necessary for viral entry and disease development, has been reported in various virus infections.^27–35^ Additionally, EGFR migration to subcellular organelles, such as ER and mitochondria, following EGFR activation, has been documented.^36–38^

In this study, we explored SARS-CoV-2-induced mitochondrial alterations influencing robust virus propagation during the early stage of infection. Our results demonstrate that SARS-CoV-2 enhances mitochondrial bioenergetics efficiency to facilitate its propagation. SARS-CoV-2 infection increases ΔΨm through the viral RNA-nucleocapsid cluster, resulting in elongated mitochondria. Furthermore, SARS-CoV-2 infection promotes oxidative phosphorylation (OXPHOS) and increases ATP production. Additionally, SARS-CoV-2 activates EGFR/Akt-mediated cell survival signalling. Subsequent EGFR translocation to the outer mitochondrial membrane (OMM) was observed via confocal microscopy and Western blot analysis using mitochondrial fractions isolated from SARS-CoV-2-infected cells and lung tissues of SARS-CoV-2-infected mice. SARS-CoV-2-induced activation of EGFR signalling and EGFR translocation to the OMM were crucial for sustaining the abnormal increase in mitochondrial OXPHOS and SARS-CoV-2 propagation. Treatment of SARS-CoV-2-infected cells with FDA-approved EGFR inhibitors resulted in a drastic reduction in SARS-CoV-2 replication and egress. Moreover, oral administration of the EGFR inhibitor vandetanib significantly suppressed SARS-CoV-2 viral load in the lung tissues of infected mice, indicating EGFR as a promising host target for combating SARS-CoV-2 propagation. Overall, our results suggest that SARS-CoV-2-induced mitochondrial alterations play a physiologically relevant role in maintaining SARS-CoV-2 persistence during the early stage of infection. Additionally, our findings offer novel insights into the antiviral potential of EGFR inhibitors as promising therapeutic agents for coronavirus disease 19 (COVID-19).

## Results

### SARS-CoV-2 aberrantly elevates mitochondrial bioenergetics

Recently, it was demonstrated that SARS-CoV-2 infects human cells through host factors ACE2 and TMPRSS2 and replicates in various human epithelial cells.^7,8^ Analysis of autopsy specimens of SARS-CoV-2-infected patients showed abundant ACE2 expression in the lung, trachea, small intestine, and kidney tissues and multiorgan infection of SARS-CoV-2.^39,40^

**Fig. 1:**
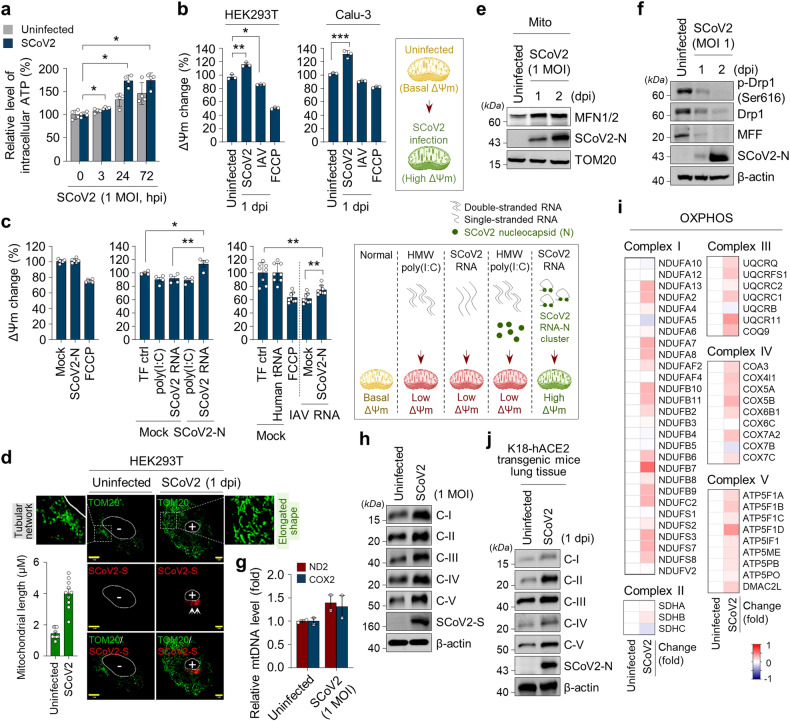
SCoV2 induces aberrant mitochondrial elongation and alterations in mitochondrial oxidative phosphorylation process. **a** Quantification of intracellular ATP level of SCoV2-infected HEK293T cells at the indicated time points (MOI of 1). Data shown are the average of two independent experiments (mean ± SD; n = 4; *p < 0.05). **b** Quantification of ΔΨ_m_ change in HEK293T (left) and Calu-3 (right) cells infected with SCoV2 and IAV (H1N1, PR8 strain), respectively (MOI of 1). ΔΨ_m_ in SCoV2 or IAV-infected cells were monitored by TMRE assay at 1 day post-infection. ΔΨ_m_ in FCCP-treated cells were monitored by TMRE assay at 2 h post-treatment. Data shown are the representative of three independent experiments (mean ± SD; n = 3; *p < 0.05, **p < 0.001, ***p < 0.0001). ΔΨ_m_, mitochondrial membrane potential. FCCP, synthetic mitochondrial uncoupler, a control for ΔΨ_m_ loss. The accompanying diagram (right) represents the SCoV2-induced increase in ΔΨ_m_. **c** Quantification of ΔΨ_m_ increase by SCoV2 RNA-N cluster. HEK293T cells were transfected with DNA plasmid encoding SCoV2 nucleocapsid (N) gene. ΔΨ_m_ in cells were monitored by TMRE assay at 24 h post-transfection (left panel). HEK293T cells transfected with DNA plasmid encoding SCoV2 nucleocapsid (N) gene for 24 h were transfected with SCoV2 RNA (1 μg/ml, middle panel), poly(I:C) (1 μg/ml, middle panel), human tRNA (1 μg/ml, right panel) and IAV RNA (1 μg/ml, right panel), respectively. ΔΨ_m_ in cells were monitored by TMRE assay at 4 h post-transfection. Data shown are the representative of three independent experiments (mean ± SD; n = 4 or n = 8; *p < 0.05, **p < 0.001). poly(I:C), synthetic analogue of double-stranded RNA, a control for ΔΨ_m_ loss; human tRNA, a control for endogenous sense RNA; TF ctrl., transfection reagent only; mock, pcDNA3.1 plasmid DNA. The accompanying diagram (right) represents the SCoV2 RNA-N cluster-induced increase in ΔΨ_m_. **d** Confocal microscopy showing the elongated shape of mitochondria in SCoV2-infected cells. SCoV2-infected HEK293T cells were immunostained with SCoV2-S (red) and TOM20 (green) antibodies. Infected (+) and uninfected (-) cells are marked. The white arrows indicate the expression of SCoV2 spike (S) antigen as an infection marker. Nuclei are demarcated with white circles. Yellow scale bar, 10 μm. The zoomed images reveal elongated shape of mitochondria in SCoV2-infected cells (right) compared with typical mitochondrial tubular network in uninfected cells (left). The accompanying graph represents the quantification of mitochondrial length by MBF ImageJ. **e** Western blot analysis of MFN1/2 expression in SCoV2-infected cells. Mitochondrial fraction (Mito) isolated from HEK293T cells infected with SCoV2 at an MOI of 1 was analysed by immunoblotting with anti-MFN1/2 antibody. SCoV2 nucleocapsid (N) protein, infection control; TOM20, an internal loading control. **f** Western blot analysis of Drp1, phospho-Drp1 (S616) and mitochondrial fission factor (MFF) expression in SCoV2-infected cells. Whole cell lysates extracted from HEK293T cells infected with SCoV2 at an MOI of 1 was analysed by immunoblotting with antibodies specific to Drp1, phospho-Drp1 (Ser616), and MFF, respectively. SCoV2 nucleocapsid (N) protein, infection control; β-actin, an internal loading control. **g** Real-time qPCR data showing the increase in mitochondrial DNA of SCoV2-infected HEK293T cells. At 1 day post-infection, the expression level of mitochondrial ND2 and COX2 DNA was analysed by real-time qPCR. GAPDH was used to normalise changes in ND2 and COX2 expression. **h** Western blot analysis of mitochondrial respiratory chain complex enzyme expression in HEK293T cells infected with SCoV2 at an MOI of 1. At 1 day post-infection, the expression level of complex I, II, III, IV and V enzymes was analysed by immunoblotting with anti-Hu total OXPHOS complex antibody. C, complex; SCoV2 spike (S) protein, infection control; β-actin, an internal loading control. **i** Heat maps of relative mRNA of the indicated mitochondrial OXPHOS genes isolated from uninfected and SCoV2-infected HEK293T cells. Each box indicates an average of three independent experiments. Colour indicates log2 fold-change for uninfected vs. SCoV2-infected cells. **j** Western blot analysis of mitochondrial respiratory chain complex enzyme expression in lung tissue isolated hACE2 transgenic mouse infected with SCoV2. At 1 day post-infection, the expression level of complex I, II, III, IV and V enzymes was analysed by immunoblotting with anti-Rodent total OXPHOS complex antibody. C, complex; SCoV2 nucleocapsid (N) protein, infection control; β-actin, an internal loading control

To investigate SARS-CoV-2-induced alterations in intracellular events, which affect rapid viral propagation during the early stage of infection, we first confirmed the abundant expression levels of endogenous ACE2 and TMPRSS2 proteins, known as key receptors for SARS-CoV-2 entry, in both human embryonic kidney 293T (HEK293T) cells and human lung epithelial Calu-3 cells through Western blot analysis (Supplementary Fig. 1a). We noted that endogenous ACE2 protein expression is suppressed in human lung epithelial A549 cells (Supplementary Fig. 1a). Next, we analysed SARS-CoV-2 infectivity and replication in HEK293T cells, Calu-3 cells and human normal primary bronchial cells by confocal microscopy (Fig. 1d, Supplementary Figs. 1b, 1f, 2a and 4a), real-time qRT-PCR (Supplementary Figs. 1c, 4b and 7b), RNAseq analysis (Fig. 3h, Supplementary Fig. 7a), Western blot analysis (Supplementary Figs. 1d, 4c and 7c), and focus forming assay (Fig. 5c, Supplementary Fig. 7d).

Interestingly, we observed a prominent elevation in intracellular ATP levels concomitant with an increase in viral replication in SARS-CoV-2-infected cells compared with uninfected cells during the early stage of infection (Fig. 1a, Supplementary Figs. 1c, 1e, 4b, 4d and 7b). To examine whether SARS-CoV-2 promotes an increase in intracellular ATP synthesis during viral replication, we measured the mitochondrial membrane potential (ΔΨm) in SARS-CoV-2-infected cells, as mitochondria are dynamic organelles known as central hub for ATP generation by oxidative phosphorylation (OXPHOS)^41,42^ and the ΔΨm is a bioenergetics parameter that modulates mitochondria respiration and is critical for mitochondrial homoeostasis.^43^ Given previous reports indicating that many viruses cause a reduction in ΔΨm,^44–47^ we found that SARS-CoV-2 infection unusually leads to an increase in ΔΨm during the early stage of infection (Fig. 1b). In contrast, our analysis of influenza A virus (IAV) revealed that IAV infection induces a loss of ΔΨm (Fig. 1b), consistent with a previous report.^47^ The accompanying schematic diagram shows that SARS-CoV-2 infection causes an increase in ΔΨm during the early stage of infection (Fig. 1b, below panel). To further strengthen our observation of the SARS-CoV-2-induced increase in ΔΨm, we conducted confocal microscopy. As shown in Supplementary Fig. 1f, our analysis revealed a significant increase in ΔΨm in SARS-CoV-2-infected cells during the early stage of infection.

To determine how SARS-CoV-2 induces ΔΨm increase, we focused on the early stage of SARS-CoV-2 infection, as most viruses trigger endoplasmic reticulum (ER) stress at the early stage of infection, leading to the release of Ca^2+^ from the ER lumen and subsequent Ca^2+^ accumulation in the mitochondrial matrix, thereby resulting in abnormal ΔΨm and mitochondrial function.^14,48^ SARS-CoV-2 ssRNA and nucleocapsid protein together enter into the cells when SARS-CoV-2 infects host cells by ACE2-mediated endocytosis.^9,10^ Hence, we sought to analyse ΔΨm in the presence of SARS-CoV-2 RNA and nucleocapsid protein. As shown in Fig. 1c, the expression of SARS-CoV-2 nucleocapsid alone did not significantly affect ΔΨm (Fig. 1c, left graph). Transfection of SARS-CoV-2 RNA in cells caused a loss of ΔΨm (middle graph), whereas its transfection in cells expressing nucleocapsid protein induced dramatic increases in ΔΨm (Fig. 1c, middle graph). Carbonilcyanide p-triflouromethoxyphenylhydrazone (FCCP) which causes collapse of ΔΨm,^49^ poly(I:C) and IAV RNA were used for the control for ΔΨm loss^50^ (Fig. 1c, middle and right graphs). Human tRNA was used for endogenous RNA control (Fig. 1c, right graph). Transfection of poly(I:C) and IAV RNA, respectively, in cells induced ΔΨm decrease in the absence of SARS-CoV-2 nucleocapsid protein, while they did not induce ΔΨm increase in the presence SARS-CoV-2 nucleocapsid protein. This finding suggests that the elaborate structure or formation of the SARS-CoV-2 ssRNA and the nucleocapsid protein might affect ΔΨm increase. The accompanying schematic diagram shows that the co-expression of SARS-CoV-2 RNA and nucleocapsid causes ΔΨm increase (Fig. 1c, right panel).

Next, we examined the mitochondrial shape using confocal microscopy, as changes in ΔΨm usually cause alterations in mitochondrial morphology, leading to abnormal mitochondrial function. As shown in Fig. 1d and Supplementary Fig. 2a, we observed that aberrantly elongated mitochondrial shapes are sustained in SARS-CoV-2-infected cells during the early stage of infection. The accompanying graph presents the quantification of mitochondrial length in SARS-CoV-2-infected cells and uninfected cells as shown in confocal microscope. Ultrastructural analysis of SARS-CoV-2-infected cells by transmission electron microscopy further substantiated the occurrence of elongated mitochondria in SARS-CoV-2-infected cells, in contrast to uninfected cells, which displayed normal mitochondrial morphology (Supplementary Fig. 2b). A quantitative analysis of relative mitochondrial length in uninfected versus SARS-CoV-2-infected cells is presented in Supplementary Fig. 2c, d. These analyses demonstrate that SARS-CoV-2 infection induces mitochondrial elongation during the early stage of infection.

Lack of mitofusin 1/2 (MFN1/2) creates mitochondrial fusion deficiency and causes severe mitochondrial fragmentation.^51^ Hence, we examined the stability of MFN1/2 in SARS-CoV-2-infected cells. As shown in Fig. 1e, we found that SARS-CoV-2 infection does not cause a reduction in MFN1/2 expression during the early stage of infection. Mitochondrial fission, crucial for cellular dynamics, relies on the intricate regulation of dynamin-1-like protein (Drp1) activation through post-translational modifications, including CDK1-induced phosphorylation at serine 616. This orchestrated process involves mediation by outer mitochondrial membrane proteins, emphasising the pivotal roles of Drp1 and mitochondrial fission factor (Mff) in the precise control of mitochondrial morphology and function.^15,41,52,53^ To further strengthen our observation of the elongated mitochondrial morphology induced by SARS-CoV-2 infection, we conducted additional analysis focused on key regulators of mitochondrial fission, encompassing the MFF, phosphorylated Drp1 (Ser616) and Drp1 itself.^15,21,24,54,55^ Our analysis revealed a noteworthy reduction in the expression levels of these constituents during the early stages of SARS-CoV-2 infection (Fig. 1f). We also demonstrated that SARS-CoV-2 induces an elevation in mitochondrial DNA (mtDNA) levels. Analysis of mitochondrial NADH dehydrogenase 2 (ND2) and cytochrome c oxidase 2 (COX2) reveals that mtDNA levels are increased in SARS-CoV-2-infected cells during the early stage of infection (Fig. 1g).

ATP is mainly generated in the mitochondria through the OXPHOS process.^56^ Thus, we subsequently examined the changes in the expression of mitochondrial OXPHOS complex that might be associated with abundant ATP production during SARS-CoV-2-induced abnormal mitochondrial fusion. We found that the expression of the mitochondrial OXPHOS complexes I, II, III, IV and V enzymes is increased in SARS-CoV-2-infected cells during the early stage of infection (Fig. 1h). RNAseq and subsequent analysis demonstrated that most genes that modulate the OXPHOS process are upregulated in SARS-CoV-2-infected cells (Fig. 1i, Supplementary Fig. 3). In addition, we observed that SARS-CoV-2 infection leads to an increase in OXPHOS complex I and V enzyme activities during the early stage of infection (Supplementary Fig. 1g, h). The SARS-CoV-2-induced increase in the expression of mitochondrial OXPHOS complexes I, II, III, IV and V enzymes was also confirmed by Western blot analysis of lung tissues of hACE2 transgenic mice infected with SARS-CoV-2 (Fig. 1j). Taken together, these results indicate that SARS-CoV-2 induces an increase in ΔΨm, mitochondrial elongation and OXPHOS process, thereby abnormally promoting ATP production.

### SARS-CoV-2 activates the EGFR signal cascade and induces mitochondrial EGFR translocation

**Fig. 2:**
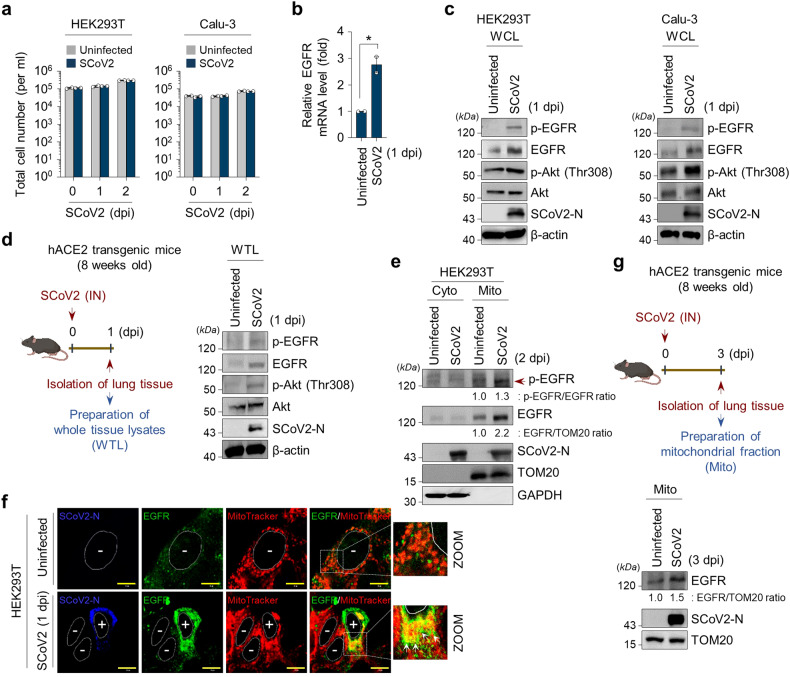
SCoV2 promotes EGFR-mediated cell survival signal cascade and mitochondrial EGFR translocation. **a** Quantification of SCoV2-infected cell viability. Total number of viable HEK293T (left) or Calu-3 (right) cells infected with SCoV2 at an MOI of 1 was measured at the indicated time points as described in Materials and Methods. Data shown are the representative of three independent experiments (mean ± SD; n = 2). **b** Quantitative analysis of EGFR gene expression in SCoV2-infected cells. At 1 day post-infection, the expression level of EGFR mRNA in HEK293T cells infected with SCoV2 at an MOI of 1 were analysed by real-time qRT-PCR (mean ± SD; n = 3; *p < 0.05). Data shown are the representative of two independent experiments. **c** Western blot analysis showing SCoV2-induced activation in EGFR-mediated signal cascade in SCoV2-infected cells. At 1 day post-infection, whole cell lysates (WCL) of HEK293T (left) or Calu-3 (right) cells infected with SCoV2 at an MOI of 1 were analysed by immunoblotting with antibodies specific to p-EGFR, EGFR, p-Akt (Thr308) and Akt. SCoV2 nucleocapsid (N) protein, infection control; β-actin, an internal loading control. **d** Western blot analysis showing SCoV2-induced activation in EGFR-mediated signal cascade in lung specimen of SCoV2-infected hACE2 transgenic mouse. At 1 day post-infection, whole tissue lysates (WTL) of lung specimens of SCoV2-infected hACE2 transgenic mouse were analysed by immunoblotting with antibodies specific to p-EGFR, EGFR, p-Akt (Thr308) and Akt. SCoV2 nucleocapsid (N) protein, infection control; β-actin, an internal loading control. **e** Western blot analysis of SCoV2-induced mitochondrial translocation of EGFR. HEK293T cells were infected with SCoV2 at an MOI of 1. At 2 days post-infection, cytosolic (Cyto) and mitochondrial (Mito) fractions isolated from uninfected and SCoV2-infected HEK293T cells were evaluated by immunoblotting with antibodies specific to p-EGFR and EGFR. Fractions: purified cytoplasm, Cyto; purified mitochondria, Mito. Organelle marker: TOM20, mitochondria; GAPDH, cytoplasm. Infection marker: SCoV2 nucleocapsid (N) antigen. The relative intensity of EGFR normalised to TOM20 and p-EGFR normalised to EGFR, respectively, was analysed by ImageJ. **f** Confocal microscopy showing mitochondrial translocation of EGFR in SCoV2-infected cells. Uninfected (upper) and SCoV2-infected (lower) HEK293T cells prestained with MitoTracker (red) were immunostained with antibodies specific to EGFR (green) and SCoV2-N (blue). Nuclei are demarcated with white circles. Infected (+) and uninfected (-) cells are marked. Yellow scale bar, 5 μm (upper), 10 μm (lower). In the zoomed images, the white arrow indicate endogenous EGFR recruited to the mitochondria in SCoV2-infected cells (yellow). **g** Western blot analysis of mitochondrial EGFR translocation in the lung tissues of hACE2 transgenic mouse infected with SCoV2. Mitochondrial fractions (Mito) isolated from lung tissue of uninfected and SCoV2-infected hACE2 transgenic mouse were analysed by immunoblotting with EGFR antibody. Organelle marker: TOM20, mitochondria. Infection marker: SCoV2 nucleocapsid (N) antigen. The relative intensity of EGFR expression was normalised to TOM20 expression

Next, we studied the survival and proliferation of human cells infected with SARS-CoV-2, which exhibit no distinct viral cytopathogenic effect during the early stage of infection while analysing SARS-CoV-2 infectivity in human cells (Fig. 2a, Supplementary Fig. 4e). Recently, Klann et al. showed SARS-CoV-2-induced activation in EGFR signalling pathway.^57^ Thus, we also sought to analyse SARS-CoV-2-induced activation in the EGFR signal cascade, as EGFR signal cascade plays a critical role in sustaining cell survival and increased cell proliferation.^37,58^ As shown in Fig. 2b, SARS-CoV-2 promoted EGFR gene expression at the transcriptional levels during the early stage of infection. Western blot analysis revealed that SARS-CoV-2 infection activates the EGFR-mediated signal cascade, as evidenced by increased levels of phosphorylated EGFR and Akt (Fig. 2c). The SARS-CoV-2-induced activation of EGFR-mediated signal cascade was also confirmed by Western blot analysis of lung tissues from hACE2 transgenic mice infected with SARS-CoV-2 (Fig. 2d). Taken together, these data indicate an association between EGFR-mediated signalling and the proliferation of SARS-CoV-2-infected cells during the early stage of infection.

Beyond its significant contribution of EGFR signal cascade required for sustaining cell survival and proliferation, EGFR migration to subcellular organelles following EGFR activation has been reported.^36–38^ Specifically, EGFR localised in the mitochondria contributes to cell survival and the regulation of mitochondrial function.^37^ Hence, we examined whether SARS-CoV-2 induces EGFR translocation to the mitochondria. We isolated the mitochondrial fraction from SARS-CoV-2-infected cells and analysed SARS-CoV-2-induced mitochondrial EGFR translocation by Western blotting with antibodies specific to phospho-EGFR and EGFR, respectively. As shown in Fig. 2e and Supplementary Fig. 5a, SARS-CoV-2 elevated mitochondrial translocation of EGFR compared to that in uninfected cells. Quantitative analysis revealed the difference in relative EGFR translocation in mitochondrial fraction of SARS-CoV-2-infected cells (Fig. 2e, Supplementary Fig. 5a). Confocal microscopy also confirmed EGFR translocation to the mitochondria of SARS-CoV-2-infected cells (Fig. 2f, Supplementary Fig. 5b).

To strengthen this observation in an in vivo animal model, we isolated the mitochondrial fraction from the lung tissues of hACE2 transgenic mice infected with SARS-CoV-2 and analysed mitochondrial EGFR translocation by Western blotting. As shown in Fig. 2g, SARS-CoV-2 infection promoted mitochondrial EGFR translocation in the lung tissue compared to that of uninfected hACE2 transgenic mice. Quantitative analysis revealed the difference in relative EGFR translocation in the mitochondrial fraction of the lung tissue of SARS-CoV-2-infected mice (Fig. 2g). Taken together, these results indicate that SARS-CoV-2 activates the EGFR signal cascade and enhances the mitochondrial localisation of EGFR during the early stage of infection.

### EGFR inhibitors exhibit potent antiviral activity against SARS-CoV-2

**Fig. 3:**
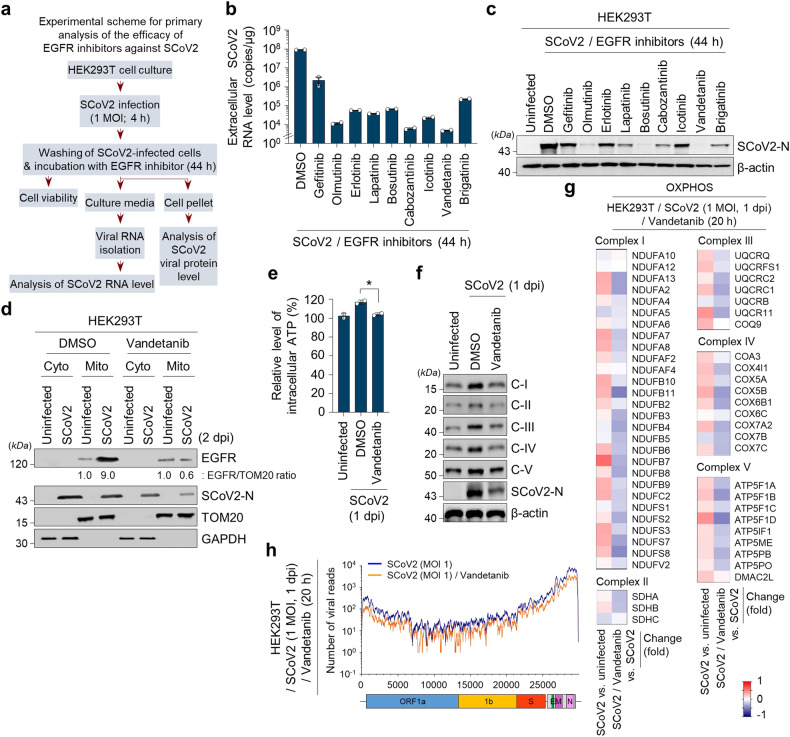
Tyrosine kinase inhibitors targeting EGFR signalling pathway reduces SCoV2 propagation. **a** A scheme for primary screening of the antiviral effect of EGFR inhibitors against SCoV2 propagation. At 4 h post-infection, HEK293T cells infected with SCoV2 at an MOI of 1 were washed with fresh cell culture media 5 times and subsequently treated with the indicated EGFR inhibitors (10 μM) for 44 h. Cell culture supernatant and pellet were used for further analyses (**b**, **c**). **b** Extracellular RNA was isolated from culture supernatants of SCoV2-infected HEK293T cells and used for the quantification of SCoV2 RNA levels by real-time qRT-PCR. Data shown are the representative of two independent experiments (mean ± SD; n = 2). **c** Whole cell lysates of SCoV2-infected HEK293T cells were analysed by immunoblotting with antibody specific to SCoV2 N protein. β-actin, an internal loading control. **d** Rescue of SCoV2-induced mitochondrial translocation of EGFR by vandetanib treatment. Cytosolic (Cyto) and mitochondrial (Mito) fractions isolated from uninfected and SCoV2-infected HEK293T cells (MOI of 1) treated with vandetanib (10 μM) were analysed by immunoblotting with EGFR antibody. Fractions: purified cytoplasm, Cyto; purified mitochondria, Mito. Organelle marker: TOM20, mitochondria; GAPDH, cytoplasm. Infection marker: SCoV2 nucleocapsid (N). **e** Rescue of SCoV2-induced elevation in intracellular ATP level by vandetanib treatment. At 1 day post-treatment of vandetanib (10 μM), intracellular ATP level in SCoV2-infected HEK293T cells (MOI of 1) was analysed as described in Materials and Methods. Data shown are the average of two independent experiments (mean ± SD; n = 2; *p < 0.05). **f**, Western blot analysis of mitochondrial respiratory chain complex enzyme expression. At 1 day post-treatment of vandetanib (10 μM), the expression level of complex I, II, III, IV and V enzymes was analysed by immunoblotting with anti-Hu total OXPHOS complex antibody. C, complex; β-actin, an internal loading control; SCoV2 N, infection marker. **g**–**h**, HEK293T cells infected with SCoV2 for 4 h at an MOI of 1 were washed with fresh cell culture media 5 times and then further cultured for 20 h in the presence of vandetanib (10 μM) for RNAseq analysis. **g** Heat maps of relative mRNA expression of the indicated mitochondrial OXPHOS genes isolated from SCoV2-infected and vandetanib-treated SCoV2-infected cells. Each box indicates an average of three independent experiments. Colour indicates log2 fold-change for SCoV2-infected vs. uninfected cells and vandetanib-treated SCoV2-infected vs. SCoV2-infected cells, respectively. **h** Read coverage across the SCoV2 genome in the presence and absence of vandetanib. The graph represents the number of viral reads per position of the SCoV2 genome in HEK293T cells (SCoV2, dark blue; SCoV2/vandetanib, orange). A scaled model of the SCoV2 genome and its genes is portrayed below

Next, we evaluated whether the robust propagation of SARS-CoV-2 is suppressed by the inhibition of SARS-CoV-2-activated EGFR signal cascade. Recent reports have shown anti-SARS-CoV-2 activity of EGFR inhibitors.^57,59^ Hence, we measured the antiviral activity of 12 FDA-approved EGFR inhibitors, including gefitinib, olmutinib, erlotinib, lapatinib, bosutinib, cabozantinib, icotinib, vandetanib, brigatinib, afatinib, dacomitinib and neratinib, in SARS-CoV-2-infected cells (Fig. 3a). As shown in Fig. 3b, treatment of SARS-CoV-2-infected cells with EGFR inhibitors caused a drastic reduction in extracellular SARS-CoV-2 RNA levels. Western blot analysis also demonstrated that treatment with EGFR inhibitors results in the reduction in the expression of the SARS-CoV-2 nucleocapsid protein (Fig. 3c). Among the 12 EGFR inhibitors studied, afatinib, dacomitinib and neratinib induced a striking cytotoxicity in SARS-CoV-2-infected cells (Supplementary Fig. 6, Supplementary Table 2). Together, these results suggest that FDA-approved EGFR inhibitors can be considered as antiviral agents for reducing the propagation of SARS-CoV-2.

### EGFR inhibitor restores SARS-CoV-2-induced abnormal mitochondrial bioenergetics

In order to substantiate the impact of EGFR inhibitors on inhibiting SARS-CoV-2 propagation, we investigated their effects on SARS-CoV-2-induced mitochondrial alterations, as we found that SARS-CoV-2 infection leads to EGFR translocation onto mitochondria (Fig. 2e–g, Supplementary Fig. 5a, b). Among the EGFR inhibitors tested, the FDA-approved vandetanib revealed the highest antiviral efficacy against SARS-CoV-2 infection (Fig. 3b, c). Hence, we examined whether vandetanib is capable of restoring SARS-CoV-2-induced abnormal OXPHOS process and the subsequent increase in ATP synthesis. We found that vandetanib inhibits mitochondrial EGFR translocation in SARS-CoV-2-infected cells (Fig. 3d, Supplementary Fig. 8a). Treatment of SARS-CoV-2-infected cells with vandetanib dramatically restored SARS-CoV-2-induced increases in intracellular ATP levels and the expression of mitochondrial OXPHOS complexes I, II, III, IV and V enzymes (Fig. 3e, f). In addition, RNAseq and subsequent analysis demonstrated that vandetanib restores SARS-CoV-2-induced abnormal upregulation in most genes that modulate the OXPHOS process, concomitant with a reduction in intracellular SARS-CoV-2 RNA levels (Fig. 3g, h, Supplementary Fig. 8b, c). We also state that treatment of SARS-CoV-2-uninfected HEK293T cells with vandetanib slightly reduces the expression of most genes involved in the OXPHOS process in our experimental condition (Supplementary Fig. 8b). Taken together, these results indicate that SARS-CoV-2-induces the activation of the EGFR signal cascade and the subsequent mitochondrial EGFR translocation, thereby contributing to SARS-CoV-2-induced abnormal mitochondrial dynamics and bioenergetics to support robust SARS-CoV-2 propagation.

### EGFR inhibitor vandetanib exhibits a potent anti-SARS-CoV-2 activity

**Fig. 4:**
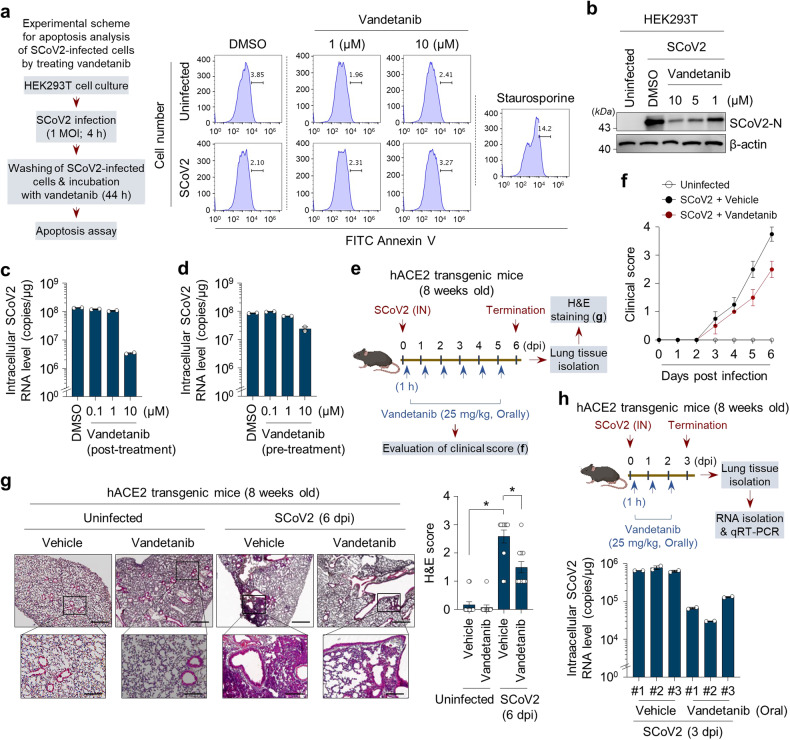
Vandetanib is a potent antiviral agent for SCoV2 propagation. **a** Apoptosis analysis of SCoV2-infected and uninfected cells treated with vandetanib. HEK293T cells infected with SCoV2 at an MOI of 1 for 4 h were further cultured in the presence of vandetanib (1 or 10 μM) for 44 h. Apoptotic cell death was analysed by flow cytometry as described in Materials and Methods. Staurosporine (200 nM, 44 h) was used as a positive control for inducing apoptotic cell death. Data shown are the representative of two independent experiments. **b** Dose-dependent antiviral effect of vandetanib against SCoV2 infection. At 2 days post-treatment, whole cell lysates of SCoV2-infected HEK293T cells (MOI of 1) treated with vandetanib at the indicated concentrations were analysed by immunoblotting with SCoV2 N antibody. β-actin, an internal loading control. **c** Anti-SCoV2 effect by post-treatment of vandetanib. HEK293T cells infected with SCoV2 at an MOI of 1 for 4 h were washed with fresh cell culture media and then treated with vandetanib (0.1, 1, or 10 μM) for 44 h. **d** Anti-SCoV2 effect by pre-treatment of vandetanib. HEK293T cells were treated with vandetanib (0.1, 1, or 10 μM) for 4 h and then infected with SCoV2 at an MOI of 1 for 44 h. Intracellular SCoV2 RNA level in SCoV2-infected cells was analysed by real-time qRT-PCR using PCR primers set specific to the SCoV2 N gene (**c**, **d**). Data shown are the representative of two independent experiments (mean ± SD; n = 2). **e**–**h**, In vivo anti-SCoV2 efficacy of vandetanib in hACE2 transgenic mouse model susceptible to SCoV2 infection. **e** A scheme for analysing clinical disease score and inflammation in SCoV-2-infected mice orally administrated with vandetanib (**f**, **g**). Eight-week-old hACE2 transgenic mice (n = 5 per group) were intranasally (IN) inoculated with SCoV2 (2 × 10^3^ pfu/head, 10 MLD_50_, clade S). One hour later, they were orally administrated with vandetanib (25 mg/kg) daily. At 6 days post-infection, all mice were terminated for further analyses (**f**, **g**). **f** Clinical scores of SCoV2-uninfected hACE2 transgenic mice (open circle), SCoV2-infected hACE2 transgenic mice administrated with vehicle (black circle) and SCoV2-infected hACE2 transgenic mice administrated with vandetanib (red circle). Vehicle control, PBS with 1% Tween 80 (**f**–**h**). Clinical scores for all mice were monitored daily based on ruffled fur (1 point), reduced mobility (1 point), hunched posture (1 point) and death (4 points) as described in Materials and Methods. **g** Immunohistochemistry analysis showing the effect of vandetanib in SCoV2-induced severe lung injury in hACE2 transgenic mouse. At 6 days post-infection, immunohistochemistry analysis was performed as described in Materials and Methods. Black scale bar, 500 μm (upper), 100 μm (lower). H&E score (right panel, mean ± SD; n = 12; *p < 0.0001). **h** In vivo anti-SCoV2 efficacy of vandetanib in hACE2 transgenic mice. Eight-week-old hACE2 transgenic mice (n = 3 per group) were intranasally (IN) inoculated with SCoV2 (2 × 10^3^ pfu/head, clade S). One hour later, they were orally administrated with vandetanib (25 mg/kg) daily. At 3 days post-infection, all mice were terminated for further analyses (upper panel). Intracellular SCoV2 RNA levels of lung tissues isolated from SCoV2-infected hACE2 transgenic mice was analysed by real-time qRT-PCR. Each data point represents the average of two independent experiments (mean ± SD; n = 2, lower panel)

To substantiate the antiviral effect of vandetanib on SARS-CoV-2 propagation, we first analysed its cell cytotoxicity. As shown in Fig. 4a, vandetanib did not significantly affect the viability of both uninfected and SARS-CoV-2-infected cells. Furthermore, vandetanib led to a dose-dependent reduction in SARS-CoV-2 nucleocapsid expression, as assessed by Western blotting (Fig. 4b). To further understand the action of the anti-SARS-CoV-2 effect of vandetanib, we analysed the expression levels of intracellular SARS-CoV-2 RNA in infected cells under post-treatment or pre-treatment of vandetanib. As shown in Fig. 4c, d, vandetanib treatment after SARS-CoV-2 infection (vandetanib post-treatment) drastically reduced intracellular SARS-CoV-2 RNA levels. In contrast, vandetanib treatment before SARS-CoV-2 infection (vandetanib pre-treatment) caused a marginal reduction in intracellular SARS-CoV-2 RNA levels, as evidenced by real-time qRT-PCR data. This suggests that vandetanib affects an intracellular replication process during SARS-CoV-2 propagation.

To further analyse the in vivo efficacy of vandetanib against SARS-CoV-2 infection, we utilised hACE2 transgenic mice. Vandetanib was initially developed as an orally available agent.^60^ Hence, we orally administrated vandetanib to SARS-CoV-2-infected hACE2 transgenic mice at a dose of 25 mg/kg daily and terminated them at 6 days post-infection to analyse clinical symptoms in hACE2 transgenic mice (Fig. 4e). As shown in Fig. 4f, we confirmed that SARS-CoV-2 infection induces severe clinical symptoms in hACE2 transgenic mice consistent with previous reports.^61,62^ We observed that SARS-CoV-2-induced severe clinical symptoms are significantly alleviated by vandetanib treatment (Fig. 4f).

SARS-CoV-2 infection caused severe lung inflammation in hACE2 transgenic mouse.^61^ Thus, we investigated whether vandetanib treatment mitigates SARS-CoV-2-induced lung inflammation. We found that SARS-CoV-2 infection induces severe lung inflammation in hACE2 transgenic mice compared to vehicle-treated controls, whereas vandetanib treatment diminishes the pathological lesions of alveolar epithelial cells and focal haemorrhage in SARS-CoV-2-infected hACE2 transgenic mice (Fig. 4g). Quantitative analysis of H&E staining is presented in the right panel of Fig. 4g. A recent report demonstrated the correlation between representative inflammatory genes, such as CCR2, CCR5, CCL5, CCR8, ARG1 and IL-10, and the development of SARS-CoV-2-induced lung inflammation in hACE2 transgenic mice.^63^ Hence, we conducted additional analyses to determine whether vandetanib treatment regulates these inflammatory genes. As a result, we observed that vandetanib treatment reduces the SARS-CoV-2-induced upregulation of expression in representative inflammatory genes, such as CCR2, CCR5, CCL5, CCR8, ARG1 and IL-10, in the lung tissues of hACE2 transgenic mice compared to those treated with the vehicle control (Supplementary Fig. 9a, b). Additionally, to analyse the effect of vandetanib on the intracellular SARS-CoV-2 RNA levels in the lung tissues of hACE2 transgenic mice, we orally administrated vandetanib to SARS-CoV-2-infected hACE2 transgenic mice at dose of 25 mg/kg daily and terminated them at 3 days post-infection (Fig. 4h). We confirmed that despite intranasal infection with a high dose (10 MLD_50_) of SARS-CoV-2, intracellular SARS-CoV-2 RNA levels in the lung tissues were significantly reduced by vandetanib treatment (Fig. 4h). Taken together, our data indicate that vandetanib exhibits potent in vitro and in vivo antiviral activity by modulating SARS-CoV-2 propagation and the SARS-CoV-2-induced inflammatory response.

**Fig. 5:**
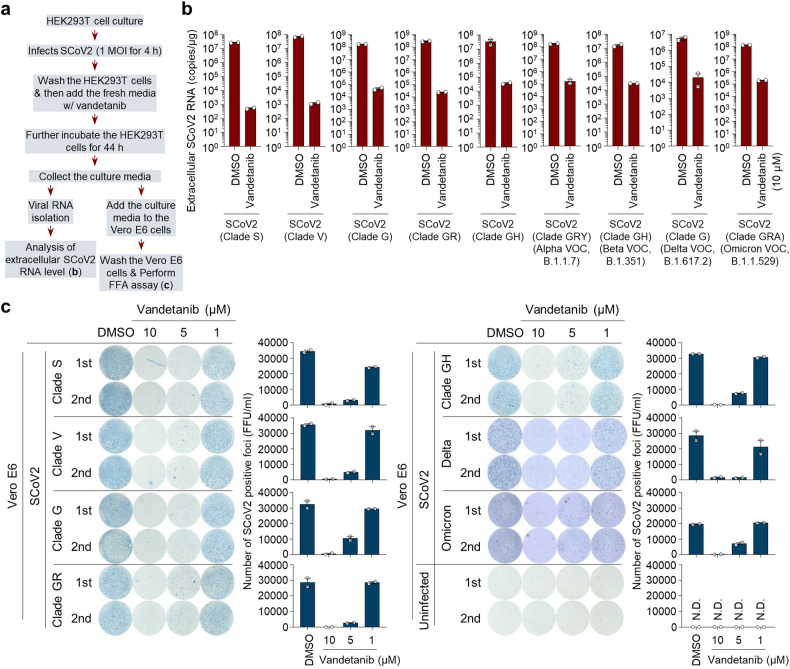
Vandetanib reveals a potent antiviral effect against various SCoV2 variants. **a** A scheme for analysing the antiviral effect of vandetanib (EGFR inhibitor) against various SCoV2 variants. HEK293T cells were infected with SCoV2 (S, V, G, GH and GR clades) and their variants of concern (VOC) including alpha (B.1.1.7), beta (B.1.351), delta (B.1.617.2) and omicron (B.1.529) variants, respectively, at an MOI of 1. At 4 h post-infection, HEK293T cells were washed with fresh cull culture media 5 times and then further incubated in the presence of vandetanib for 44 h. Cell culture media was used for further analyses of real-time qRT-PCR (**b**) and FFU assay using Vero E6 cells (**c**). **b** Real-time qRT-PCR data showing reduced extracellular SCoV2 RNA levels following treatment with vandetanib. Total RNA was isolated from the culture media of HEK293T cells infected with SCoV2 in the presence of vandetanib (10 μM) and then used for analysis of extracellular SCoV2 RNA level by real-time qRT-PCR using PCR primers set specific to the SCoV2 N gene. Data shown are the representative of two independent experiments (mean ± SD; n = 2). DMSO was used as the negative control. **c** FFU assay data showing the reduction in SCoV2 infectivity following treatment with vandetanib. Cell culture media of SCoV2-infected HEK293T cells post-treated with vandetanib at the indicated concentrations (1, 5 and 10 μM) were transferred to fresh Vero E6 cells and then further incubated for 8 h for FFU assay as described in Materials and Methods. SCoV2, cell culture media of SCoV2-infected HEK293T cells in the presence of vandetanib; Uninfected, cell culture media of SCoV2-uninfected HEK293T cells. The accompanying graphs show the average of two independent experiments (right panel). DMSO was used as the negative control. N.D. not determined

Recently, SARS-CoV-2 variants has been constantly spreading worldwide. Thus, we examined whether vandetanib exhibits antiviral activity against various SARS-CoV-2 lineages (S, V, G, GH and GR clades) and its variants of concern (VOC), including B.1.1.7 (alpha variant), B.1.351 (beta variant), B.1.617.2 (delta variant) and B.1.1.529 (omicron variant). The anti-SARS-CoV-2 activity of vandetanib was evaluated by qRT-PCR analysis and SARS-CoV-2 focus forming assay (FFA) (Fig. 5a). As shown in Fig. 5b, treatment of SARS-CoV-2 variant-infected cells with vandetanib caused a drastic reduction in the expression levels of extracellular SARS-CoV-2 RNA. Furthermore, vandetanib potently inhibited the infectivity of various SARS-CoV-2 strains in a dose-dependent manner (Fig. 5c). The SARS-CoV-2 strain-induced foci formation was completely inhibited at 10 μM of vandetanib (Fig. 5c). Taken together, these results suggest that vandetanib is an efficient inhibitor against various SARS-CoV-2 strains and variants.

## Discussion

Since the declaration of the COVID-19 pandemic in March 2020, the World Health Organization (WHO) officially announced its end in May 2023. However, despite this declaration, SARS-CoV-2 infection remains a global threat and our understanding of persistent SARS-CoV-2 infection and its pathogenesis remains incomplete. In this study, we report that SARS-CoV-2 alters intracellular events and abnormally modulates mitochondrial bioenergetics for robust virus propagation during the early stages of infection.

While some reports have indicated a decrease in ΔΨm induced by the SARS-CoV-2 M protein.^64^ or by infection with live, whole SARS-CoV-2 virus in human hepatoma Huh7 cells or Vero E6, an interferon response-attenuated cell line,^65^ our findings reveal that SARS-CoV-2 increases ΔΨm through the SARS-CoV-2 RNA and nucleocapsid complex (Fig. 1b, c), leading to mitochondrial elongation (Fig. 1d, Supplementary Fig. 2a) during the early stages of infection. Furthermore, we demonstrated the increase of ΔΨm in human lung epithelial Calu-3 cells and HEK293T cells infected with SARS-CoV-2, as evidenced by the expression of the SARS-CoV-2 nucleocapsid protein through confocal microscopy (Supplementary Fig. 1f). Interestingly, Díaz-Resendiz et al. showed a decrease in mitochondrial membrane potential (MMP) in human PBMCs isolated from patients infected with SARS-CoV-2 and in subjects recovered from infection at various time points.^66^ This data is informative for understanding changes in mitochondria in recovered SARS-CoV-2 patients and the development of long-COVID-19. However, it’s important to consider that mitochondrial dynamics and MMP alteration can be influenced by various stress conditions besides virus infection. Moreover, we found that SARS-CoV-2 infection activates the mitochondrial oxidative phosphorylation (OXPHOS) process to generate abundant ATP during the early stages of infection. SARS-CoV-2-induced alterations in the OXPHOS process might be alternatively explained by different mechanisms, including SARS-CoV-2 NSP1-mediated translational shutdown.^67–69^

Previously, Cortese et al. reported the presence of thinner mitochondria in SARS-CoV-2-infected cells.^70^ In this study, we observed both thinner (in a horizontal orientation) and numerous elongated (in a longitudinal orientation) mitochondria in SARS-CoV-2-infected cells during the early stages of infection, as evidenced by transmission microscopy (Supplementary Fig. 2b–d). This observation suggests that SARS-CoV-2 infection induces alterations in mitochondrial morphology, encompassing both thinner and elongated structures when compared to uninfected cells.

**Fig. 6:**
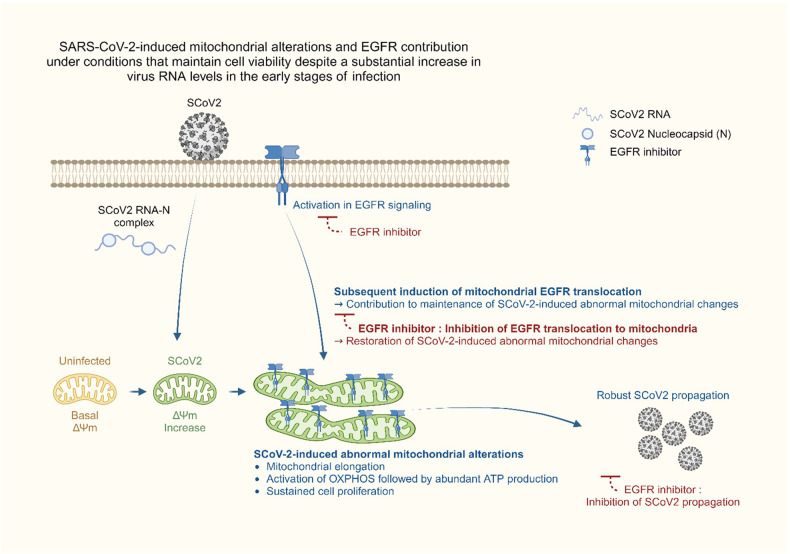
A schematic diagram showing the significant contribution of SCoV2-induced altered mitochondrial dynamics and mitochondrial EGFR translocation in sustaining viral propagation. First, SCoV2 RNA and nucleocapsid complex increase ΔΨm during the early stages of SCoV2 infection. This alteration subsequently promotes mitochondrial elongation. SCoV2 also activates the mitochondrial OXPHOS process, thereby promoting ATP production. Second, SCoV2 activates EGFR-mediated cell survival signalling and subsequently promotes mitochondrial EGFR internalisation, which contributes to the maintenance of abnormal mitochondrial bioenergetics. These alterations are physiologically relevant to the maintenance of homoeostasis of SCoV2-infected cells and robust SCoV2 propagation

EGFR has been reported as a co-factor for numerous virus entry processes, and the involvement of EGFR internalisation and transport to cellular organelles provides a favourable environment for virus replication.^27–31,71–74^ In this study, we showed that SARS-CoV-2 stimulates EGFR-mediated cell survival signal during the early stages of infection (Figs. 2b–d) and subsequently promotes mitochondrial EGFR translocation (Figs. 2e–g, Supplementary Fig. 5), contributing to the maintenance of abnormal mitochondrial bioenergetics. These findings were demonstrated by the analyses of subcellular fractions and confocal microscopy. Subsequently, SARS-CoV-2 maintained the abnormally elevated mitochondrial bioenergetics via EGFR internalisation on the mitochondria, affecting viral replication and ATP production. Overall, our results suggest that SARS-CoV-2-modulated abnormal mitochondrial bioenergetics is physiologically relevant to the maintenance of cellular homoeostasis in SARS-CoV-2-infected cells and robust SARS-CoV-2 replication and propagation (Fig. 6).

Ligand-stimulated EGFR trafficking pathways involve autophosphorylation, signalling at the plasma membrane and subsequent internalisation into endosomes, with potential routes to the ER and other organelles in response to stressors.^38,75^ During virus-induced stress responses, such as ER stress and oxidative stress, cellular organelles undergo reorganisation.^76^ This alteration may potentially lead to the internalisation of EGFR on various membranes, including endosomes, the ER, etc. While our study demonstrates EGFR internalisation onto mitochondria during the early stages of SARS-CoV-2 infection, the comprehensive exploration of EGFR translocation to reorganised intracellular organelles, such as endosomes and the ER, remains an avenue for future investigation.

In hepatitis C virus (HCV), EGFR acts as a co-factor for entry and upregulates its internalisation and transport to the cellular organelles; similar processes have been also reported in hepatitis B virus (HBV) and gastroenteritis virus.^27–32,77^ SARS-CoV infection also involved the overactivation of EGFR, which leads to increased pulmonary fibrosis.^57,78^ Of note, many respiratory viruses induce EGFR activation in the airway epithelium.^31,79^ Moreover, the aberrant expression and activation of EGFR have been implicated in multiple diseases, including cancer, diabetes, pulmonary fibrosis and various lung, heart and renal diseases.^33,58,80–82^

Notably, in this study, vandetanib caused a remarkable inhibition of SARS-CoV-2 propagation against all clades of SARS-CoV-2. Vandetanib, an FDA-approved EGFR inhibitor, is an inexpensive drug with a low toxicity and has been used for the treatment of certain tumours of the thyroid gland.^83,84^ Several EGFR inhibitors have been shown to block multiple steps of replication in influenza virus^85^ and dengue virus.^86^ Therapeutic options against the various SARS-CoV2 strains are limited. In the context of the current COVID-19 pandemic, our findings suggest that EGFR inhibitors act as a druggable regulator that reduces viral replication by modulation of mitochondrial bioenergetics and the EGFR signal cascade against SARS-CoV-2 variants.

Three years after the onset of the COVID-19 pandemic, only a limited number of antiviral therapies have emerged, providing viable options for early administration in individuals at risk of severe disease, as well as interventions to improve outcomes in cases of advanced disease. These therapeutic approaches can be broadly categorised as direct-acting antivirals and host-directed therapies. Both strategies target specific viral components or host cell components required for viral replication, as well as those that mitigate the dysregulated inflammatory response to infection. Effective administration of these therapies early during infection, before the virus reaches its replication peak, is crucial and serves to prevent progression to severe disease.^87–90^ Our study underscores the potential of EGFR inhibitors in inhibiting the propagation of SARS-CoV-2 during the early stages of infection (Figs. 3–5, Supplementary Fig. 9). The findings suggest that EGFR inhibitors may represent promising candidates for antiviral drug development. Originally formulated as EGFR inhibitors for lung cancer, these drugs, exemplified by vandetanib, may demonstrate efficacy for lung cancer patients concurrently grappling with SARS-CoV-2-induced diseases. This dual-purpose application underscores the repurposing potential of existing drugs, illustrating a synergistic approach to addressing.

## Materials and methods

### Cell culture

Human embryonic kidney 293 T cells (American Type Culture Collection, ATCC, CRL-3216) and human lung epithelial A549 cells (ATCC) were grown in high-glucose DMEM (Gibco) containing 1% nonessential amino acids (Gibco), 1% sodium pyruvate (Gibco) and 10% foetal bovine serum (FBS) (Hyclone). Human lung epithelial Calu-3 cells (ATCC) were cultured in DMEM/F12 (Gibco) supplemented with 10% FBS. Human normal primary bronchial cells (ATCC) were grown in bronchial epithelial basal medium (LONZA). All cell lines were incubated in a 5% CO_2_ humidified atmosphere at 37 °C.

### Viruses

SARS-CoV-2 variants were kindly provided by the Korea Centers for Disease Control and Prevention. The variants used in this study are listed in Supplementary Table 1.^91^ SARS-CoV-2 was propagated and prepared in African green monkey kidney Vero E6 cells (ATCC). SARS-CoV-2 infection was performed at a multiplicity of infection (MOI) of 1 in a biosafety level 3 (BSL3) facility. All experiments using SARS-CoV-2 was handled in an enhanced BSL3 containment laboratory as approved by the Korea Centers for Disease Control and Prevention.

### Reagents and antibodies

The US Food and Drug Administration (FDA)-approved EGFR inhibitors, including gefitinib, olmutinib, erlotinib, lapatinib, bosutinib, cabozantinib, icotinib, vandetanib, brigatinib, afatinib, dacomitinib and neratinib as well as remdesivir were provided by the Korea Chemical Bank at the Korea Research Institute of Chemical Technology (Daejeon, Republic of Korea). The primary antibodies used in this study included the following: rabbit monoclonal anti-phospho-EGFR (Y1068, Y1092) (Abcam, #40815), rabbit polyclonal anti-EGFR (Millipore, #06-847), rabbit monoclonal anti-phospho-Akt (T308) (Cell Signalling Technology, #13038), rabbit monoclonal anti-Akt (Cell Signalling Technology, #4691), mouse monoclonal anti-MFN1/2 (Abcam, #57602), rabbit monoclonal anti-Drp1 (Cell Signalling Technology, #8570), rabbit monoclonal anti-phospho-Drp1 (S616) (Cell Signalling Technology, #3455), rabbit monoclonal anti-MFF (Cell Signalling Technology, #84580), mouse monoclonal anti-β-actin (Cell Signalling Technology, #3700S), mouse monoclonal anti-TOM20 (BD, #612278), rabbit monoclonal anti-GAPDH (Cell Signalling Technology, #2118), mouse monoclonal anti-Hu total OxPhos complex (Thermo Fisher Scientific, #45-8119), mouse monoclonal anti-Rodent total OxPhos complex (Abcam, #110413), rabbit polyclonal anti-ACE2 (Abcam, #15348), mouse monoclonal anti-TMPRSS2 (SantaCruz, #515727), rabbit monoclonal anti-SARS-CoV-2 nucleoprotein (Sino Biological, #40143-R019) and rabbit polyclonal anti-SARS-CoV-2 spike (Sino Biological, #40150-T62-COV2). The secondary antibodies used for immunofluorescence were Alexa Fluor 350, 488, or 647 donkey anti-mouse, rabbit, or goat IgG (Life Technologies, #10039, #11055, #31571). The secondary antibodies used for western blot analyses were HRP-conjugated anti-mouse IgG (Invitrogen, Waltham, MA, USA, #62-6520) and HRP-conjugated anti-rabbit IgG (Invitrogen, #31460).

### Western blot analysis

For the immunoblotting assay, whole cell lysates were prepared by adding cell lysis buffer (20 mM Tris-HCl [pH 7.5], 150 mM NaCl, 1 mM EDTA, 1 mM DTT, 50 mM NaF, 1 mM Na_3_VO_4_, 0.5% TritonX-100, 1% SDS, 1 mM β-glycerophosphate and protease inhibitor cocktail). Proteins were separated via SDS-PAGE and transferred onto PVDF membranes (Millipore) that were blocked with 5% skim milk in PBS containing 0.1% Tween-20 (PBS-T) and stained with antibodies against the indicated proteins. The membranes were washed three times with PBS-T and developed using the western ECL Femto Kit (LPS Solution). Images were captured using ImageQuant LAS 4000 (GE Healthcare). The intensity of protein expression was quantified using ImageJ (Bethesda).

### Tetramethylrhodamine, ethyl ester assay

A mitochondrial membrane potential assay kit (BioVision) was used according to the manufacturer’s instructions. Total cells were incubated with the fluorescent tetramethylrhodamine, ethyl (TMRE, 200 nM) dye for 20 min at 37 °C and 5% CO_2_. For the negative control, FCCP (carbonyl cyanide 4-(trifluoromethoxy) phenylhydrazon, 20 μM) was added into one well and incubated at 37 °C for 10 min prior to TMRE addition. Following incubation, the stained cells were harvested and gently washed with the assay buffer. Fluorescence signal was measured at 549/575 nm of excitation/emission wavelengths using a Synergy H1 multi-mode microplate reader (BioTek). Two technical and biological replicates were performed for each sample.

### Real-time qRT-PCR and qPCR

To analyse the expression levels of EGFR and SARS-CoV-2 viral genes, total RNA was extracted from cells using an RNeasy Mini Kit (Qiagen) as per the manufacturer’s instruction. Extracellular viral RNA from supernatants was isolated using a QIAamp Viral RNA Mini Kit (Qiagen). The cellular RNA levels of *EGFR, Ccr2, Ccl5, Arg1, Ccr5, Ccr8* and *IL-10* were quantified by real-time qRT-PCR using a One-Step SyBr Green RT-PCR Kit II (Takara). The intracellular and extracellular SARS-CoV-2 RNA copy numbers were determined by real-time qRT-PCR using a One Step PrimeScript III RT-PCR Kit (Takara). The following primer sets were used for RT-PCR: EGFR forward, 5′-AGGCAGGAGTAACAAGCTCAC; EGFR reverse, 5′-ATGAGGACATAACAAGCCACC; CCR8 forward, 5′-AGTGGGCAGCTCTGAAAC, CCR8 reverse, 5′-GCTCCATCGTGTAATCCATCG, IL-10 forward, 5′-AGCCGGGAAGACAATAACTG, IL-10 reverse, 5′-GGAGTCGGTTAGCAGTATGTTG, ARG1 forward, 5′-AAGAATGGAAGAGTCAGTGTGG, ARG1 reverse, 5′-GGGAGTGTTGATGTCAGTGTG, CCR2 forward, 5′-GCTCTACATTCACTCCTTCCAC, CCR2 reverse, 5′-ACCACTGTCTTTGAGGCTTG, CCL5 forward, 5′-GGGTACCATGAAGATCTCTGC, CCL5 reverse, 5′-TCTAGGGAGAGGTAGGCAAAG, CCR5 forward, 5′-TCCAGCAAGACAATCCTGATC, CCR5 reverse, 5′-AACCATTCCTACTCCCAAGC, SARS-CoV-2-N forward, 5′-TTACAAACATTG GCCGCAAA; SARS-CoV-2-N reverse 5′-GCGCGACATTCCGAAGAA; SARS-CoV-2-N probe 5′-FAM-ACAATTTGCCCCCAGCGCTTCAG-BHQ1; β-actin forward, 5′-ACAGAGCCTCGCCTTTG; β-actin reverse, 5′-CCTTGCACATGCCGGAG, and β-actin probe; 5′-/56-FAM/TCATCCATG/ZEN/GTGAGCTGGCGG/3IABkFQ.

To analyse the expression levels of mitochondrial DNA, total cellular DNA was extracted from the cells using an AllPrep DNA kit (Qiagen) and quantified by qPCR using a DyNAmo HS SYBR Green qPCR kit according to the manufacturer’s instructions. The following primer sets were used for qPCR: ND-2 forward, 5′-TAGCCCCCTTTCACTTCTGA; ND-2 reverse, 5′-GCGTAGCTGGGTTTGGTTTA; COX-2 forward, 5′-GGCCACCAATGGTACTGAAC; COX-2 reverse, 5′-CGGGAATTGCATCTGTTTTT, GAPDH forward, 5′-GCCATCAATGACCCCTTCATT; and GAPDH reverse, 5′-TTGACGGTGCCATGGAATTT. Real-time qPCR was conducted on the QuantStudio3 Real-Time PCR System (Applied Biosystems).

### Immunofluorescence

Cells grown on glass cover slips were infected with SARS-CoV-2 at an MOI of 1. At 1 or 2 days post-infection, cells were fixed with 4% paraformaldehyde, permeabilized with 0.2% Triton X-100 in phosphate-buffered saline (PBS), blocked with blocking buffer (2% BSA, PBS) and immunostained with the indicated antibodies. MitoTracker CMXRos Red (Invitrogen) was used to stain mitochondria in live cells before fixation. The coverslips were mounted in antifade medium (Vector Laboratories). Images were captured using Olympus Fluoview FV3000 confocal laser-scanning microscope and evaluated using Olympus FV31S-SW.

### Cell viability assay

Cell viability was measured by the CellTiter-Glo assay (Promega) using a Synergy H1 microplate reader (BioTek) according to the manufacturer’s instructions. The total cell number and cell viability were quantified using a haemocytometer and the Trypan Blue exclusion method.

### ATP assay

Intracellular ATP levels were measured with the ATP assay kit (Abcam, Cat#: ab83355) using a Synergy H1 microplate reader (BioTek) following the manufacturer’s instructions.

### Measurement of mitochondrial complex I and V enzyme activity

The activity of mitochondrial oxidative phosphorylation respiratory chain Complex I and Complex V in SARS-CoV-2-infected cells was measured using the Mitochondrial Complex I enzyme activity assay kit (Abcam, Cat# ab109721) and the Mitochondrial Complex V (ATP Synthase) enzyme activity assay kit (Abcam, Cat# ab109714), following the manufacturer’s instructions. Briefly, Calu-3 cells were infected with SARS-CoV-2 for the indicated time points (3, 24 and 72 hpi, respectively) and detergent-soluble whole cell lysates were then directly utilised for these assays.

### Subcellular fractionation

Mitochondria were isolated from SARS-CoV-2-infected cells or lung tissues of SARS-CoV-2-infected mice using a mitochondria isolation kit (Thermo Scientific, Cat#: 89874 for cultured cells, Cat#: 89801 for tissue) according to the manufacturer’s instructions. Protein extracted from each cellular fraction was analysed by western blotting with the indicated antibodies.

### Animal experiments

Mouse experiments were approved by the Institutional Animal Care and Use Committee (IACUC) of the Korea Research Institute of Chemical Technology (approval number 2020-8B-07-01). Nine K18-hACE2 transgenic mice (8 weeks old) were evenly divided into three groups and housed in the ABSL-3 facility. Mice from each group were lightly euthanized with isoflurane and intranasally inoculated with SARS-CoV-2 (2 × 10^3^ pfu/head, clade S). Mice were orally administered vehicle (PBS with 1% Tween 80) or vandetanib (25 mg/kg per mouse) once daily. Mouse lung samples were washed with PBS and harvested to determine the inhibition of SARS-CoV-2. Tissue samples from the lung were homogenised in cold PBS solution with a FastPrep-24 homogeniser (MP Biomedicals) for five cycles (20 s on/20 s off), followed by three additional freeze–thaw cycles were performed at −80 °C. Cell debris was removed by centrifugation at 13,000 rpm for 1 min to measure the viral RNA and proteins in the lung.

### Histopathological analysis

For the haematoxylin and eosin (H&E) histopathological analysis, lung tissues were dissected and fixed in 10% neutral buffered formalin at room temperature. The fixed tissues were processed by dehydration and embedded in paraffin. Each embedded tissue was sectioned into 4 µm thickness sections. Lung tissue sections were stained with H&E for light microscopy examination. The degree of lung damage was assessed by the degeneration of alveolar epithelial cells, broadening of alveolar septa and infiltration of inflammatory cells. The criteria used to assess the histological grading of lung damage were previously described.^62,92^ The degree of alveolar epithelial cell degeneration was assessed by a histological grading scale as follows: score of 0 when alveolar epithelial cells degeneration was not observed, 1 when the degeneration of alveolar epithelial cells was less than 10% of the total cells, 2 when the degeneration was 10–30% and 3 when the degeneration was more than 30%. To evaluate the degree of the broadening of alveolar septa, we scored 0 when there was no broadening, 1 when broadening was observed in less than 10% of the total cells, 2 when broadening was 10–30% and 3 when broadening was more than 30%. The following histological grading was used to evaluate the degree of inflammatory cell infiltration: score of 0 when no inflammatory cell infiltration was visible; 1 when the region of inflammatory cell infiltration was less than 10%, 2 when the region was 10–30%, and 3 when the region reached more than 30%. The total score per animal was calculated by the sum of the score of the severity from four sections.

### Focus-forming unit assay

The titre of infectious virus in the culture medium was determined by the focus-forming unit (FFU) assay. Vero E6 cells were seeded in a 96-well plate. The infectious cell culture medium was used to infect the cells and the plates were incubated at 37 °C. After 8 h, cells were washed with PBS, fixed with 4% paraformaldehyde and permeabilized with methanol. SARS-CoV-2 foci were developed using anti-SARS-CoV-2 N antibody, followed by addition of horseradish peroxidase-conjugated goat anti-rabbit IgG secondary antibody and TMB substrate (Promega). The plates were incubated in dark condition at room temperature for 30 min, washed and dried for observation under a light microscope. The number of SARS-CoV-2 positive foci was scanned and counted in each well using an ImmunoSpot reader (CTL, Shaker Height, OH).

### RNAseq analysis and data processing

Raw reads were mapped to the human genome assembly hg38 using the aligner STAR v.2.4.0b.^93^ To measure gene expression, the gene annotation database of the species was used with Cufflinks v2.1.1.^94^ For differential expressed gene (DEG) analysis, the gene level count data were generated by HTSeq-count v0.6.1p.^95^ The R package TCC.^96^ was used to identify DEGs. To compare tag count data, this package applies robust normalisation strategies. Normalisation factors were measured using the iterative DEGES/edgeR method. To correct errors due to multiple testing, the DEGs were identified based on the *q*-value threshold < 0.05.^97^

### Transmission Electron Microscopy

HEK293T cells infected with SARS-CoV-2 at an MOI of 10 were prepared for electron microscopy. At 1 day post-infection, cells were fixed and embedded as previously described.^98^ Briefly, after washing with 0.1 M phosphate buffer, cells were post-fixed with 1% osmium tetroxide for 2 h at 4 °C. For dehydration, cells underwent an ethanol series (50%, 70%, 75%, 90%, 95% and 100%) and were subsequently placed in propylene oxide. Dehydrated cells were embedded in Embed-812 resins (Electron Microscopy Sciences, EMS) and polymerised at 70 °C for 24 h. They were sectioned at 80 nm using a Leica Ultramicrotome (Leica, Bensheim, Germany) with diamond knives and mounted on 150 mesh copper grids. The sectioned cells were post-stained with 2% uranyl acetate and 1% lead citrate. Finally, the cells were observed using conventional TEM (JEM-1400 Plus, JEOL, Tokyo, Japan) at 120 kV.

### Apoptosis analysis

For the apoptosis analysis of SARS-CoV-2-infected cells induced by vandetanib treatment, an apoptosis assay was conducted using the Annexin V Apoptosis Detection Kit (eBioscience) following the manufacturer’s instructions. Briefly, SARS-CoV-2-infected HEK293T cells (5 × 10^5^ cells) in the presence or absence of vandetanib were resuspended in binding buffer, followed by staining with Annexin V and PI for 15 min. The fluorescence signals of all samples were then analysed using the BD Accuri C6 plus flow cytometer (BD Biosciences). Staurosporine (200 nM, 44 h) was used as a positive control for inducing apoptotic cell death.

### Statistical analysis

Unpaired Student’s *t*-tests and one-way ANOVA were performed using GraphPad Prism 8 (Graph Pad, La Jolla, CA, USA).

### Supplementary information

Supplementary Material

Dataset 1

Dataset 2

## Data Availability

All data generated or analysed during this study are included in this published article and its supplementary information files or are available from the corresponding author upon reasonable request. Source Data file is provided with this paper.
