# Supplementary material for: SARS-CoV-2 aberrantly elevates mitochondrial bioenergetics to induce robust virus propagation
Source: Signal Transduct Target Ther. 2024 May 11;9:125. doi: 10.1038/s41392-024-01836-x (PMC11088672; doi:10.1038/s41392-024-01836-x)
Supplement: Supplementary file 1 — Supplementary Material [file 41392_2024_1836_MOESM1_ESM.pdf]

# Supplementary Materials for

## SARS-CoV-2 aberrantly elevates mitochondrial bioenergetics to induce robust virus propagation

Hye Jin Shin[1,#], Wooseong Lee[1], Keun Bon Ku[1], Gun Young Yoon[1], Hyun-Woo Moon[1], Chonsaeng Kim[1], Mi-Hwa Kim[1,2], Yoon-Sun Yi[3], Sangmi Jun[3], Bum-Tae Kim[1], Jong-Won Oh[4], Aleem Siddiqui[5], and Seong-Jun Kim[1,*]

Correspondence to: sekim@krict.re.kr (S.-J. K.)

**This PDF file includes:**

Supplementary Figures. S1 to S9

Supplementary Tables. 1 to 2

## Supplementary Figures

### Supplementary Fig. 1

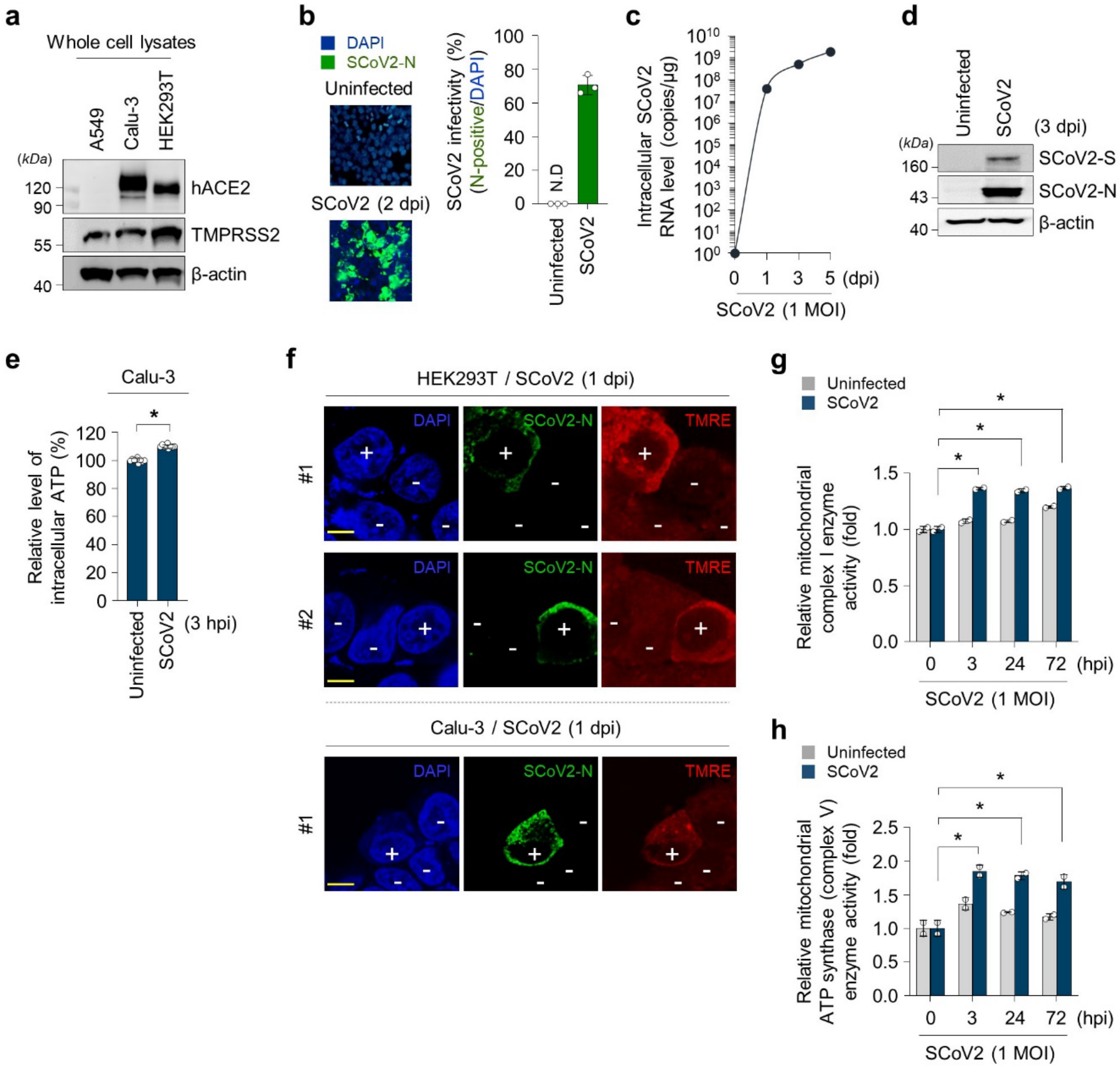


**Supplementary Fig. S1 | SCoV2 infection leads to the elevation in intracellular ATP level, mitochondrial complex I and V enzyme activities, and mitochondrial membrane potential ($\Delta\Psi_m$). a-d**, SCoV2 infectivity and replication in HEK293T cells. **a**, Western blot analysis showing the expression of endogenous ACE2 and TMPRSS2 genes in A549, Calu-3, and HEK293T cells. Whole cell lysates of A549, Calu-3, and HEK293T cells resuspended in lysis buffer containing 1% SDS were analysed with antibodies specific to ACE2 and TMPRSS2. β-actin was used an internal loading control. **b**, Immunofluorescence microscopic analysis of SCoV2 infectivity in HEK293T cells. HEK293T cells infected with SCoV2 at an MOI of 1 were immunostained with SCoV2-N

antibody (green) at 2 days post-infection. Immunofluorescent signal was analysed by Operetta microscope. Nuclei, DAPI (blue); infection marker, SCoV2 nucleocapsid (N) antigen (green). The accompanying graph shows the quantification of SCoV2-N-positive cells. **c**, Real-time qRT-PCR data showing a rapid increase in intracellular SCoV2 RNA level of HEK293T cells. Data shown are the average of two independent experiments. **d**, Western blot data showing the expression of SCoV2 spike and N antigens in SCoV2-infected HEK293T cells. HEK293T cells were infected with SCoV2 at an MOI of 1 and then further cultured for 3 days. Whole cell lysates of SCoV2-infected HEK293T cells were analysed by immunoblotting with antibodies specific to SCoV2 spike and N antigens. S, spike protein; N, SCoV2 nucleocapsid; β-actin, an internal loading control. **e**, Quantification of SCoV2-induced an increase in intracellular ATP levels. Calu-3 cells were infected with SCoV2 at an MOI of 1. At 3 hours post-infection, intracellular ATP level was analysed as described in Materials and Methods (mean ± SD; n=9; *p<0.001). **f**, Representative confocal microscopy showing $\Delta\Psi_m$ increase in SCoV2-infected cells. HEK293T (upper panel) and Calu-3 (lower panel) cells were infected with SCoV2 at an MOI of 1. At 1 day post-infection, SCoV2-infected cells prestained with TMRE dye (red) were immunostained with SCoV2-N antibody. $\Delta\Psi_m$ in SCoV2-infected cells were analysed by confocal microscope using TMRE staining. Nuclei, DAPI (blue); infection marker, SCoV2 nucleocapsid (N) antigen (green); $\Delta\Psi_m$, mitochondrial membrane potential. Infected (+) and uninfected (-) cells are marked. Yellow scale bar, 10 μm. Data shown are the representative images of independent experiments using HEK293T (#1 and #2) and Calu-3 (#1) cells, respectively. **g-h**, Quantification of mitochondrial complex I (**g**) and V (**h**) enzyme activities increased by SCoV2 infection. Calu-3 cells were infected with SCoV2 at an MOI of 1 and were subsequently used for further assays. At the indicated time points, the activity of mitochondrial complex I and V enzymes was measured according to manufacturer's instructions (mean ± SD; n=2; *p<0.01).

**Supplementary Fig. 2**

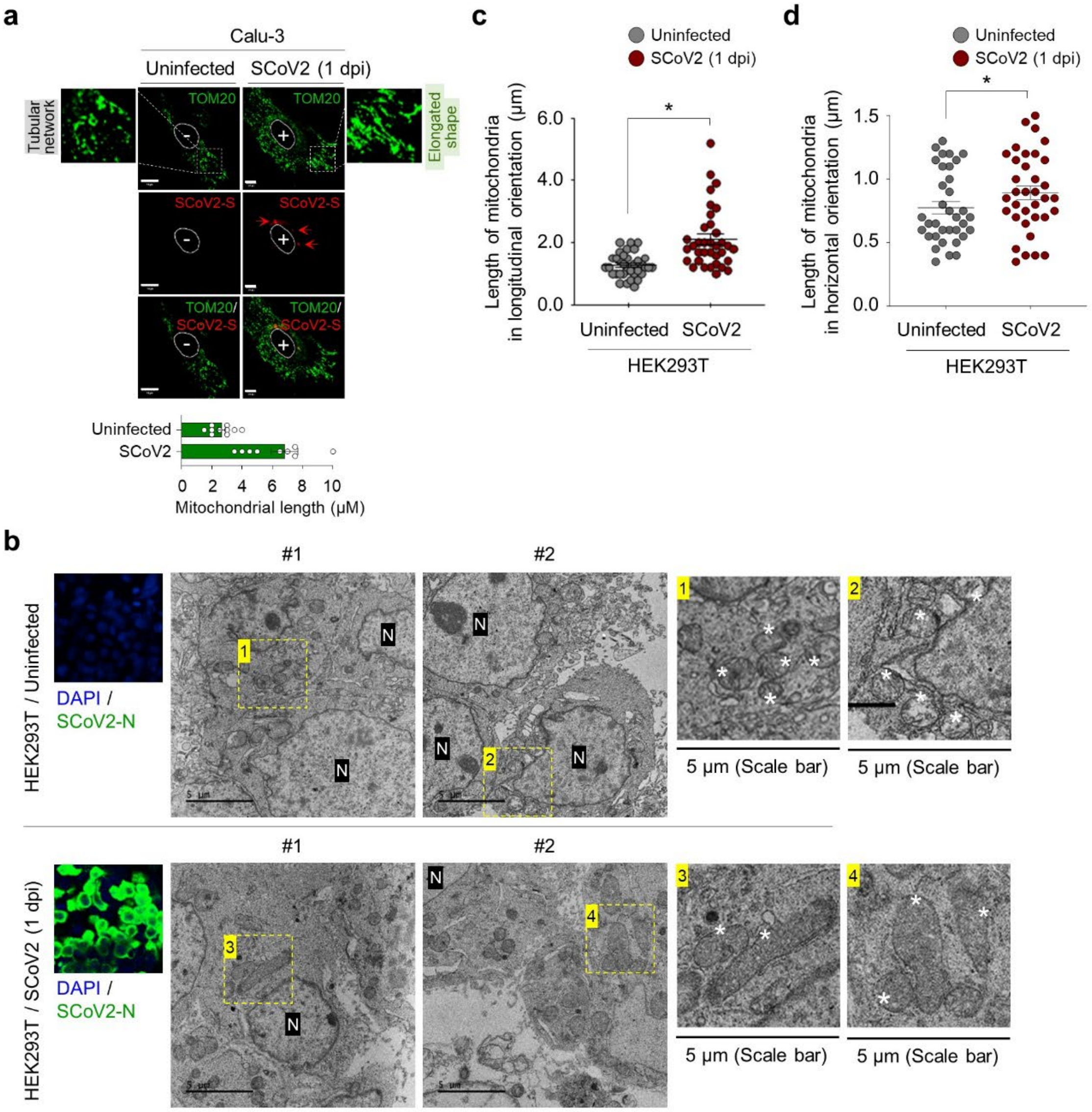


**Supplementary Fig. S2 | SCoV2 infection induces aberrant mitochondrial elongation. a**, Confocal microscope image of the elongated mitochondria of SCoV2-infected Calu-3 cells. SCoV2-infected Calu-3 cells were immunostained with antibodies specific to SCoV2-S (red) and TOM20 (green). Infected (+) and uninfected (-) cells are marked. The red arrows indicate the expression of SCoV2 spike (S) antigen as an infection marker. Nuclei are demarcated with white circles. White scale bar, 10 µm. The zoomed images reveal elongated shape of mitochondria in SCoV2-infected cells (right) compared to typical mitochondrial tubular network in uninfected cells (left). The accompanying graph represents the quantification of mitochondrial length by MBF ImageJ. **b**, Transmission electron microscopy of SCoV2-infected HEK293T cells showing the

elongated mitochondria. HEK293T cells were infected with SCoV2 at an MOI of 10. At 1 day post-infection, the ultrastructure of uninfected and SCoV2-infected HEK293T cells was examined by transmission electron microscope as described in Materials and Methods. In the zoomed images, normal mitochondria in uninfected cells (#1 and #2, upper panel) and elongated mitochondria in SCoV2-infected cells (#1 and #2, lower panel) are shown. Black scale bar, 5 μm. Organelle marker: N, nucleus. Confocal images: SCoV2 N, green; nuclei, blue (DAPI). **c-d**, MBF ImageJ quantification of mitochondrial length (**c**, longitudinal orientation; **d**, horizontal orientation) shown in **b**.

**Supplementary Fig. 3**

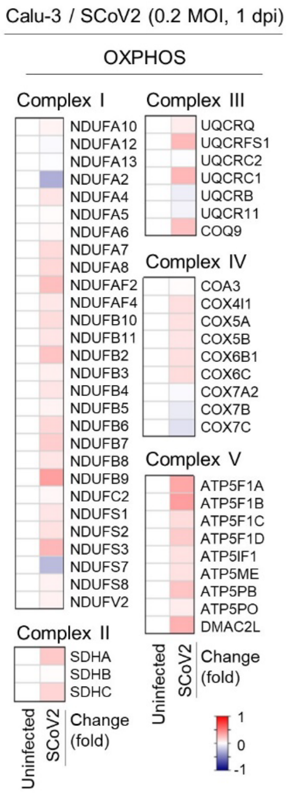


**Supplementary Fig. S3 | SCoV2 infection elevates the expression of OXPHOS genes.** Heat maps of the relative expression of the indicated OXPHOS genes in uninfected and SCoV2-infected Calu-3 cells. Calu-3 cells infected with SCoV2 for 1 day at an MOI of 0.2 were used for analysis of the expression level of OXPHOS genes by RNAseq as described in Materials and Methods. Each box indicates an average of three independent experiments. Colour indicates log2 fold-change for SCoV2-uninfected vs -infected cells.

**Supplementary Fig. 4**

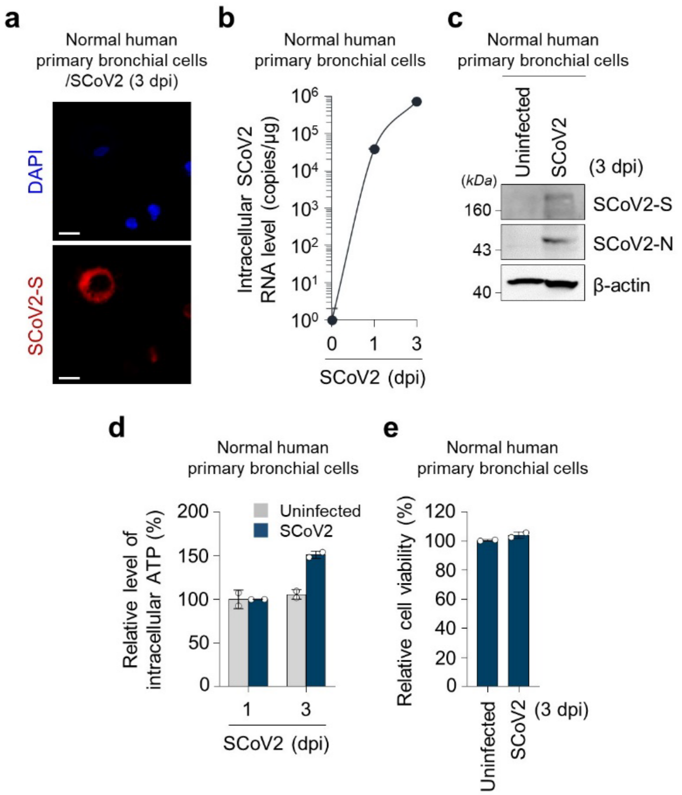


**Supplementary Fig. S4 | SCoV2 infection leads to the elevation in intracellular ATP synthesis in normal human primary bronchial cells during the early stages of SCoV2 infection. a**, Confocal microscopy showing SCoV2 infectivity in normal human primary bronchial cells. At 3 days post-infection, SCoV2-infected normal human primary bronchial cells were immunostained with SCoV2 spike antibody (red). Nuclei, DAPI (blue). White scale bar, 10 μm. **b**, Real-time qRT-PCR data showing a rapid increase in intracellular SCoV2 RNA levels in normal human primary bronchial cells. Normal human primary bronchial cells infected with SCoV2 for the indicated time points at an MOI of 1 were used for analysis of the expression level of intracellular SCoV2 RNA by real-time qRT-PCR as described in Materials and Methods. Data shown are the average of two independent experiments. **c**, Western blot data showing the expression of SCoV2 spike and N antigens in SCoV2-infected normal human primary bronchial cells. Normal human primary bronchial cells infected with SCoV2 for 3 days at an MOI of 1 were used for analysis of the expression level of SCoV2 spike and N antigens by Western blot assay. Whole cell lysates of SCoV2-infected normal human primary bronchial cells were analysed by immunoblotting with

antibodies specific to SCoV2 spike and N antigens. S, SCoV2 spike; N, SCoV2 nucleocapsid; β-actin, an internal loading control. **d**, Quantification of increased intracellular ATP levels of SCoV2-infected normal human primary bronchial cells. Normal human primary bronchial cells infected with SCoV2 for the indicated time points at an MOI of 1 were used for analysis of the intracellular ATP level as described in Materials and Methods. Data are representative of two independent experiments. **e**, Cell viability of SCoV2-infected normal human primary bronchial cells. At 3 days post-infection, total number of normal human primary bronchial cells infected with SCoV2 was measured by using a hemocytometer and trypan blue.

Supplementary Fig. 5

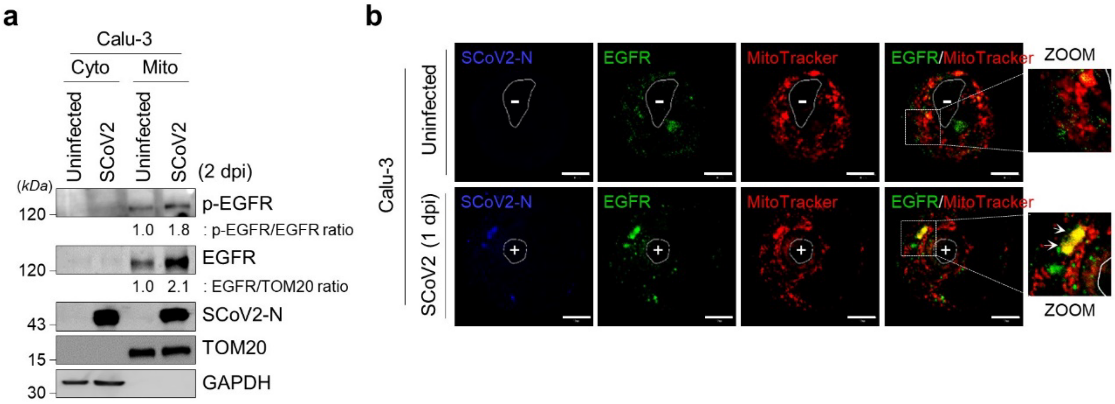


**Supplementary Fig. S5 | SCoV2 promotes mitochondrial EGFR translocation. a**, Western blot analysis showing SCoV2-induced EGFR translocation on mitochondria. Calu-3 cells were infected with SCoV2 at an MOI of 1. At 2 days post-infection, cytosolic (Cyto) and mitochondrial (Mito) fractions isolated from uninfected and SCoV2-infected Calu-3 cells were evaluated by immunoblotting with antibodies specific to p-EGFR and EGFR. Organelle marker: TOM20, mitochondria; GAPDH, cytoplasm. Infection marker: SCoV2 nucleocapsid (N) antigen. The relative intensity of EGFR normalized to TOM20 and p-EGFR normalized to EGFR, respectively, was analysed by ImageJ. **b**, Confocal microscopy showing mitochondrial translocation of EGFR in SCoV2-infected cells. Uninfected (upper panel) and SCoV2-infected (lower panel) Calu-3 cells prestained with MitoTracker (red) were immunostained with antibodies specific to EGFR (green) and SCoV2-N (blue). Nuclei are demarcated with white circles. Infected (+) and uninfected (-) cells are marked. White scale bar, 10 μm. In the zoomed images, the white arrow indicate endogenous EGFR recruited to the mitochondria in SCoV2-infected cells (yellow).

**Supplementary Fig. 6**

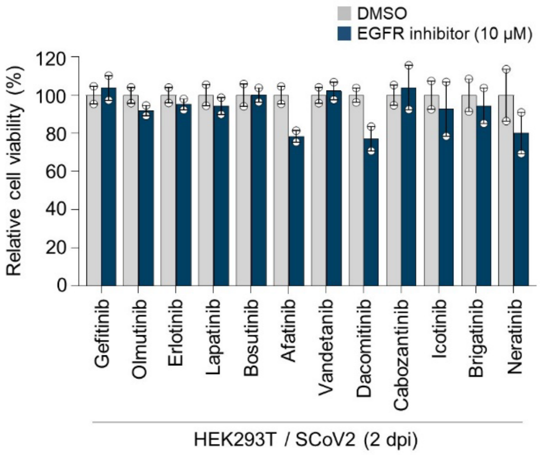


**Supplementary Fig. S6 | Cytotoxicity analysis of EGFR inhibitors in SCoV2-infected cells.** HEK293T cells infected with SCoV2 for 4 h at an MOI of 1 were washed with fresh cell culture media 5 times and then further cultured for 44 h in the presence of EGFR inhibitors (10 μM). Cytotoxicity of the SCoV2-infected HEK293T cells was analysed by CellTiter-Glo luminescent cell viability assay as described in Materials and Methods. DMSO was used as the negative control.

Supplementary Fig. 7

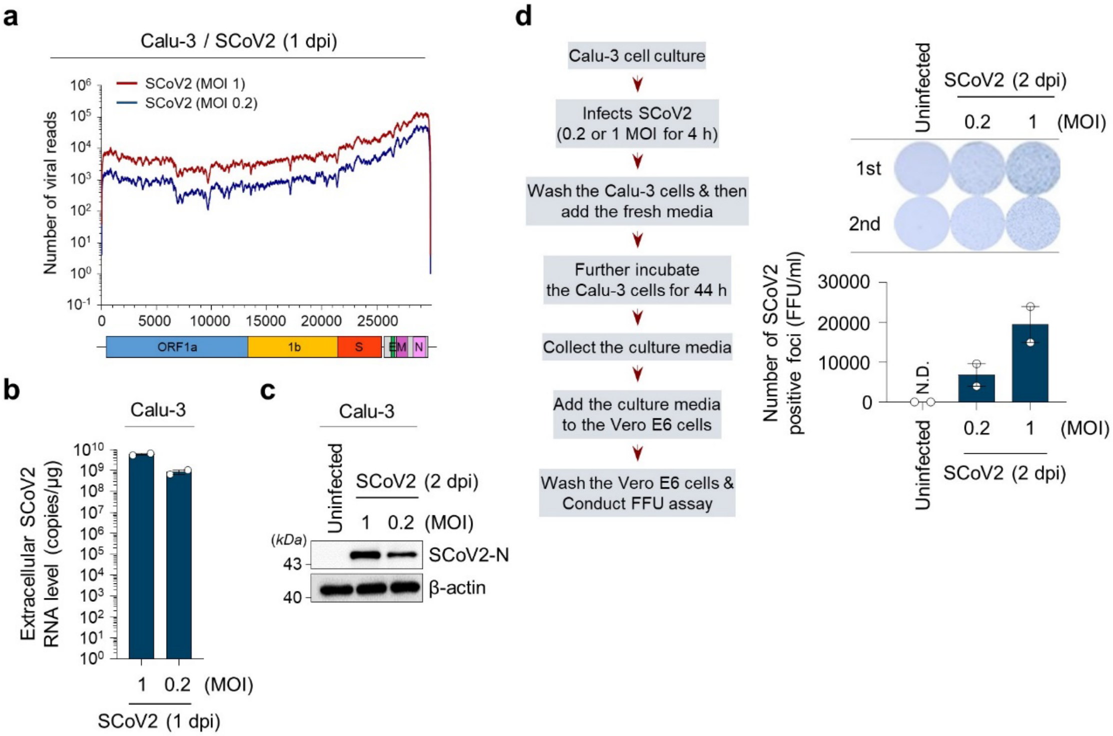


**Supplementary Fig. S7 | Analysis of SCoV2 infectivity at both low and high MOI conditions.** **a**, Read coverage across the SCoV2 genome at an MOI of 0.2 and 1, respectively. Calu-3 cells infected with SCoV2 for 1 day at an MOI of 0.2 and 1, respectively were used for RNAseq analysis. The graph represents the number of viral reads per position of the SCoV2 genome in Calu-3 cells (MOI of 0.2, dark blue; MOI of 1, dark red). A scaled model of the SCoV2 genome and its genes is portrayed below. **b**, Real-time qRT-PCR data showing extracellular SCoV2 RNA levels. Culture media of Calu-3 cells infected with SCoV2 for 1 day at an MOI of 0.2 and 1, respectively were used for real-time qRT-PCR analysis. **c**, Western blot data showing the expression level of SCoV2 N antigen in SCoV2-infected Calu-3 cells. Calu-3 cells were infected with SCoV2 at an MOI of 0.2 and 1, respectively, and then further cultured for 2 days. Whole cell lysates of SCoV2-infected Calu-3 cells were analysed by immunoblotting with antibody specific to SCoV2 antigen. N, SCoV2 nucleocapsid; β-actin, an internal loading control. **d**, (Left panel) A scheme for analysing SCoV2 infectivity at both low and high MOI conditions. Calu-3 cells were infected with SCoV2 (clade S) at an MOI of 0.2 and 1, respectively. At 4 h post-infection, Calu-3 cells were washed with fresh cull culture media 5 times and then further incubated for 44 h. Culture media of SCoV2-infected Calu-3 cells was used for FFU assay using Vero E6 cells. (Right panel) FFU assay data

showing the SCoV2 infectivity in Calu-3 cells at an MOI of 0.2 and 1, respectively. Culture media of Calu-3 cells infected with SCoV2 at the indicated concentrations (0.2 or 1 MOI) were transferred to fresh Vero E6 cells and further incubated for 8 h for FFU assay. SCoV2, cell culture media of SCoV2-infected Calu-3 cells; Uninfected, cell culture media of SCoV2-uninfected Calu-3 cells. The accompanying graph shows the average of two independent experiments (lower panel). N.D., not determined.

**Supplementary Fig. 8**

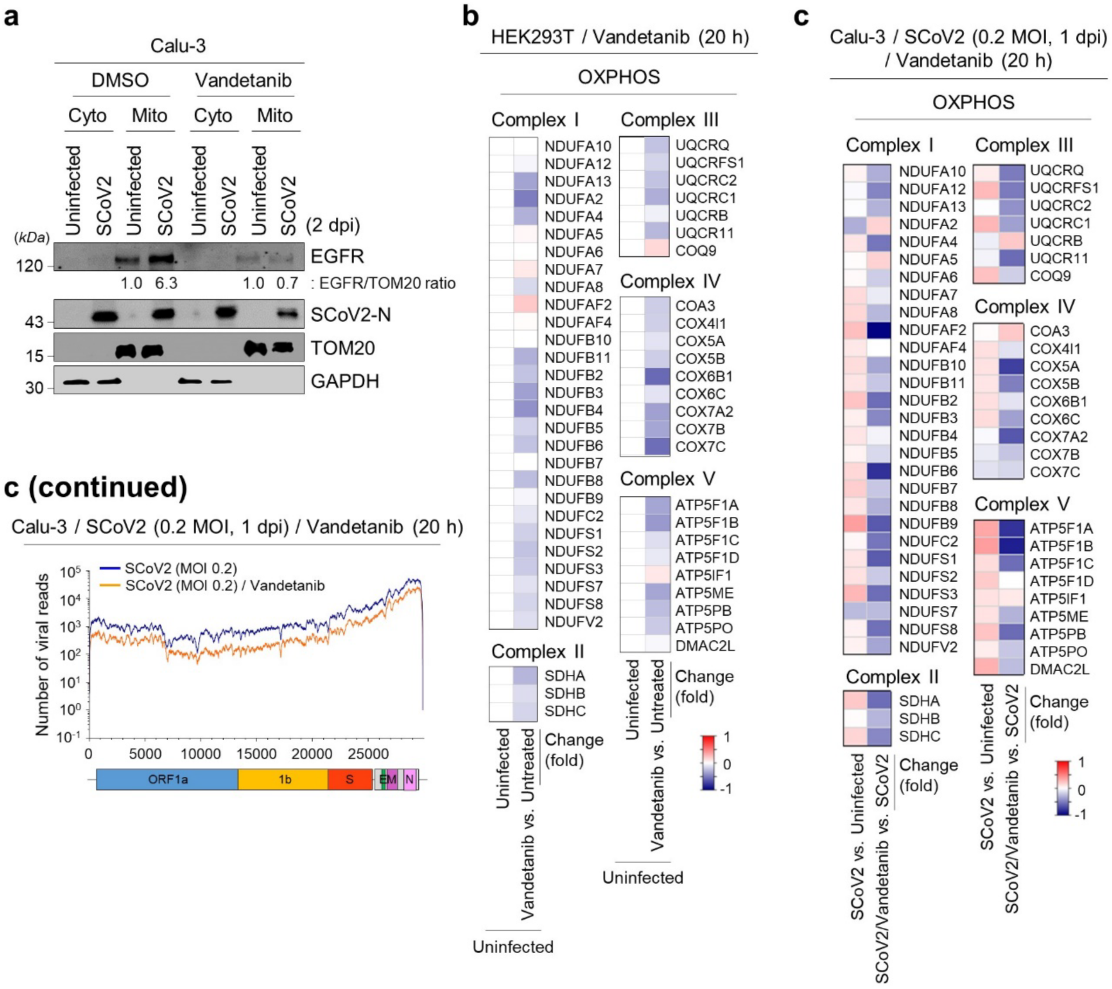


**Supplementary Fig. S8 | Vandetanib rescues SCoV2-induced abnormal mitochondrial EGFR translocation and increase in genes modulating oxidative phosphorylation (OXPHOS). a**, Rescue of SCoV2-induced abnormal mitochondrial translocation of EGFR by vandetanib treatment. Calu-3 cells infected with SCoV2 for 4 h at an MOI of 1 were washed with fresh cell culture media 5 times and then treated with vandetanib for 44 h. Cytosolic (Cyto) and mitochondrial (Mito) fractions isolated from uninfected and SCoV2-infected Calu-3 cells in the presence and absence vandetanib were analysed by immunoblotting with EGFR antibody. Organelle marker: TOM20, mitochondria; GAPDH, cytoplasm. Infection marker: SCoV2 nucleocapsid (N). DMSO was used as the negative control. **b**, Heat maps of relative mRNA expression of the indicated mitochondrial OXPHOS genes isolated from untreated (DMSO-treated control) and vandetanib-treated uninfected cells. HEK293T cells were cultured for 20 h in the

presence or absence of vandetanib for RNAseq analysis. DMSO was used as a control for vandetanib treatment. Each box indicates an average of three independent experiments. Colour indicates log2 fold-change for untreated vs. untreated cells and vandetanib-treated vs. untreated cells, respectively. **c**, Heat maps of relative mRNA expression of the indicated mitochondrial OXPHOS genes isolated from SCoV2-infected and vandetanib-treated SCoV2-infected cells. Calu-3 cells infected with SCoV2 for 4 h at an MOI of 0.2 were washed with fresh cell culture media 5 times and then further cultured for 20 h in the presence of vandetanib. SCoV2-infected Calu-3 cells were used for RNAseq analysis. Each box indicates an average of three independent experiments. Colour indicates log2 fold-change for SCoV2-infected vs. SCoV2-uninfected cells and vandetanib-treated SCoV2-infected vs. SCoV2-infected cells, respectively. The accompanying graph shows the read coverage across the SCoV2 genome in the presence and absence of vandetanib. The graph represents the number of viral reads per position of the SCoV2 genome in Calu-3 cells (SCoV2, dark blue; SCoV2/vandetanib, orange). A scaled model of the SCoV2 genome and its genes is portrayed below.

**Supplementary Fig. 9**

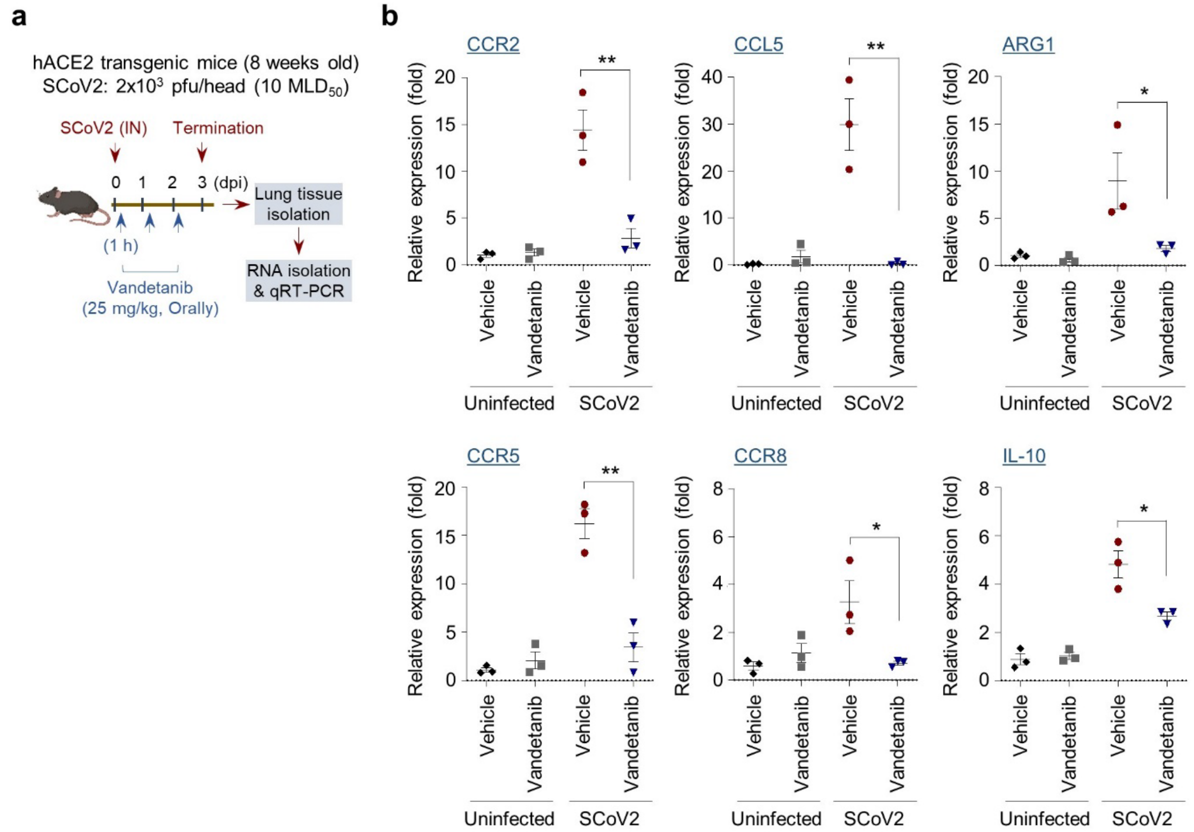


**Supplementary Fig. S9 | Vandetanib reduces SCoV2-induced transcriptional activation of pro-inflammatory genes. a**, A scheme for analysing the effect of vandetanib in hACE2 transgenic mice infected with SCoV2. Eight-week-old hACE2 transgenic mice (n=3 per group) were intranasally (IN) inoculated with SCoV2 ($2\times10^3$ pfu/head, 10 $MLD_{50}$, clade S). One hour later, they were orally administrated with vandetanib (25 mg/kg) daily. At 3 days post-infection, all mice were terminated for further analyses. **b**, Real-time qRT-PCR analysis of the indicated inflammatory genes (*CCR2, CCL5, ARG1, CCR5, CCR8,* and *IL-10*) in the lung tissues isolated form SCoV2-uninfected hACE2 transgenic mice orally administrated with vehicle or vandetanib and SCoV2-infected hACE2 transgenic mice orally administrated with vehicle or vandetanib. Vehicle control, PBS with 1% Tween 80. Each point represents an individual mouse. Data were normalized to β-actin expression in each sample (mean ± SD; n=3; $*p<0.01$; $**p<0.001$).

## Supplementary Tables

**Supplementary Table 1. SARS-CoV-2 viruses used in this study**

| Virus | Clade* | WHO label | NCCP** number | Cell line for virus propagation | Virus stock concentration (pfu/ml)*** |
|---|---|---|---|---|---|
| SARS-CoV-2 | S | | 43326 | Vero E6 | $1 \times 10^6$ |
| | V | | 43342 | | $5 \times 10^5$ |
| | G | | 43343 | | $1 \times 10^6$ |
| | GR | | 43344 | | $1 \times 10^6$ |
| | GH | | 43345 | | $1 \times 10^6$ |
| | GRY | Alpha variant (B.1.1.7) | 43381 | | $1 \times 10^7$ |
| | GH | Beta variant (B.1.351) | 43382 | | $1 \times 10^7$ |
| | G | Delta variant (B.1.617.2) | 43390 | | $1.6 \times 10^7$ |
| | GRA | Omicron variant (B.1.1.529) | 43408 | | $6 \times 10^5$ |

*SARS-CoV-2 variants used in this study: All main and supplementary figures, S clade; Fig. 5, S, V, G, GR, GH, GRY (Alpha variant), GH (Beta variant), G (Delta variant), and GRA (Omicron variant) clades.

**NCCP: National Culture Collection for Pathogens, National Institute of Health, Republic of Korea.

***After virus propagation using Vero E6 cells, virus concentration was titrated by plaque assay.

**Supplementary Table 2. Summary of FDA-approved EGFR inhibitors showing anti-SARS-CoV-2 efficacy**

| EGFR inhibitors (TKIs) | Structure | Final concentration for primary screening (μM) | Cytotoxicity* | Anti-SARS-CoV-2 efficacy** |
|---|---|---|---|---|
| Gefitinib | 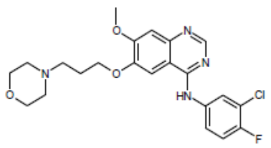 | 10 | –<br>(negative) | $\geq 4.1\times10^{1}$<br>RNA*** |
| Olmutinib | 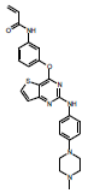 | 10 | –<br>(negative) | $\geq 7.6\times10^{3}$<br>RNA*** |
| Erlotinib | 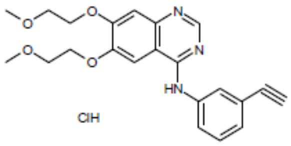 | 10 | –<br>(negative) | $\geq 1.6\times10^{3}$<br>RNA*** |
| Lapatinib | 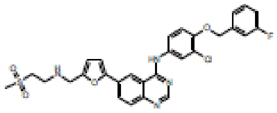 | 10 | –<br>(negative) | $\geq 2.4\times10^{3}$<br>RNA*** |
| Bosutinib | 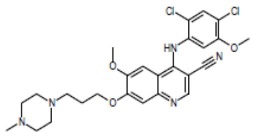 | 10 | –<br>(negative) | $\geq 1.4\times10^{3}$<br>RNA*** |
| Cabozantinib | 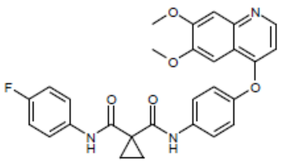 | 10 | –<br>(negative) | $\geq 1.4\times10^{4}$<br>RNA*** |
| Icotinib | 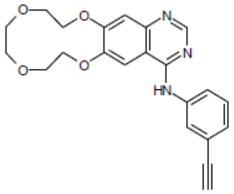 | 10 | –<br>(negative) | $\geq 4.0\times10^{3}$<br>RNA*** |

| | | | | |
|---|---|---|---|---|
| Vandetanib | 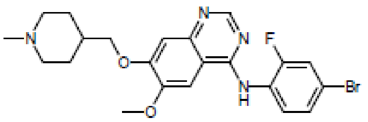 | 10 | – (negative) | ≥ $1.9\times10^4$ RNA*** |
| Brigatinib | 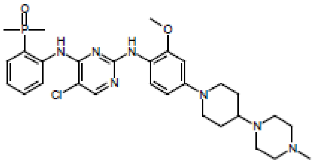 | 10 | – (negative) | ≥ $4.1\times10^2$ RNA*** |
| Afatinib | 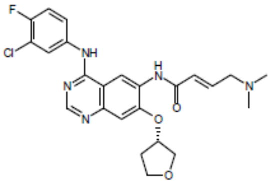 | 10 | + (positive) | N.D.**** |
| Dacomitinib | 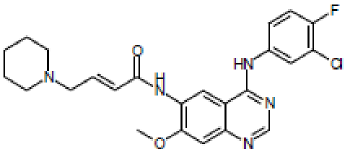 | 10 | + (positive) | N.D.**** |
| Neratinib | 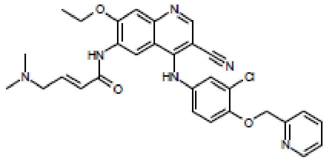 | 10 | + (positive) | N.D.**** |

* In the presence of EGFR inhibitors for 44 h, the cytotoxicity of SARS-CoV-2-infected HEK293T cells was observed by light microscope in a biosafety laboratory 3.

** The anti-SARS-CoV-2 efficacy of EGFR inhibitors was determined by real-time quantitative RT-PCR analysis of extracellular SARS-CoV-2 RNA using a primer set specific to the SARS-CoV-2 N gene. Mean: differences between non-treated vs. treated EGFR inhibitor.

*** Unit: copies/μg

**** N.D.: Not determined
